# 2-O, 3-O Desulfated Heparin (ODSH) Preserves Endothelial Barrier Integrity in Lipopolysaccharide-Induced Acute Lung Injury by Modulating TLR4–MyD88–NF-κB Signaling

**DOI:** 10.3390/biom16101516

**Published:** 2026-10-18

**Authors:** Rahul S. Patil, Shweta R. Patil, Liselle Simon, Peter Biggs, Latika Jaiswal, Basmah Mohamed, Thomas P. Kennedy, Joyce Gonzales, Alexander D. Verin

**Affiliations:** 1 Vascular Biology Center, Medical College of Georgia, Augusta University, Augusta, GA 30912, USA; rapatil@augusta.edu (R.S.P.); shwpatil@augusta.edu (S.R.P.);; 2 Cantex Pharmaceuticals Inc., Weston, FL 33326, USA; 3 GlycoMira Therapeutics, Salt Lake City, UT 84108, USA; 4 Department of Medicine, Division of Pulmonary Medicine, Wake Forest University, Winston-Salem, NC 27157, USA; 5 Division of Pulmonary, Critical Care, and Sleep Medicine, Medical College of Georgia, Augusta University, Augusta, GA 30912, USA

**Keywords:** 2-O, 3-O desulfated heparin, acute lung injury, cytokines, endothelial permeability, lipopolysaccharide

## Abstract

Bacterial lipopolysaccharide (LPS) disrupts endothelial barrier stability by inducing inflammatory events that underlie acute lung injury (ALI). Effective pharmacologic therapies capable of modifying the disease mechanisms of ALI remain limited. We previously demonstrated that 2-O, 3-O desulfated heparin (ODSH), a modified heparin with minimal anticoagulant activity and anti-inflammatory properties, attenuates LPS-induced ALI in mice and preserves human lung microvascular endothelial cell (HLMVEC) barrier integrity. In the present study, we further investigated the mechanisms of ODSH-mediated protection in mice and HLMVECs, focusing on inflammatory mediator expression and the modulation of TLR4–MyD88–NF-κB signaling. HLMVECs were pretreated with ODSH before LPS exposure, and barrier function was monitored using transendothelial electrical resistance measurements. Complementary experiments were performed in CD-1 mice receiving intratracheal LPS with or without intravenous ODSH. Across both models, ODSH attenuated LPS-induced endothelial barrier dysfunction. ODSH significantly reduced bronchoalveolar lavage fluid (BALF) protein accumulation, inflammatory cell infiltration, IL-6 and TNF-α concentrations, Evans Blue dye extravasation, and pulmonary edema. Gene expression analyses demonstrated that ODSH suppressed multiple pro-inflammatory mediators and inflammatory signaling components, including IL-1β, TNF-α, IL-6, NOS2, MAPK14, MAPK1, ICAM1, and CCL2, while enhancing IL-10 and several barrier-associated genes (CDH5, OCLN, CLDN5, and TJP1). ODSH also reduced the mRNA expression of TLR4, MYD88, and RELA. Protein analyses confirmed a reduced expression of NF-κB, MyD88, and TNF-α. Furthermore, ODSH at concentrations of 5–100 μg/mL did not significantly affect cellular metabolic activity. Collectively, these findings indicate that ODSH preserves endothelial barrier function and attenuates inflammatory responses associated with TLR4–MyD88–NF-κB signaling in experimental ALI. The findings support the further mechanistic and preclinical investigation of ODSH in inflammatory lung injury.

## 1. Introduction

Acute lung injury (ALI) and its severe form, acute respiratory distress syndrome (ARDS), arise from diverse clinical insults, including pneumonia, sepsis, trauma, and aspiration, and they are major causes of respiratory failure in critically ill patients [1]. They are characterized by extensive inflammation, disruption of the alveolar–capillary barrier, and protein-rich pulmonary edema that leads to severe hypoxemia [2,3,4]. Despite advances in supportive care such as lung-protective ventilation, prone positioning, and conservative fluid management, effective pharmacologic therapies capable of modifying the underlying disease mechanisms remain unavailable. Consequently, mortality in patients with moderate-to-severe ARDS remains approximately 30–50% [5,6].

Lipopolysaccharide (LPS), a major component of Gram–negative bacterial (e.g., *Escherichia coli*) cell walls, is widely used to model ALI in mice because it triggers potent innate immune activation in endothelial and immune cells [7,8]. The engagement of LPS with CD14, LPS-binding protein, and Toll-like receptor 4 (TLR4) initiates NF-κB- and MAPK-dependent signaling cascades that amplify cytokine production, promote neutrophil recruitment, and compromise endothelial barrier integrity [9]. Although this model reflects key pathophysiologic features of human ALI, debate persists regarding the relative contributions of endothelial versus leukocyte pathways to barrier failure, highlighting the need for mechanistic studies that address these divergent hypotheses. Endothelial barrier disruption is a central pathogenic feature of ALI/ARDS and is regulated by complex signaling networks that govern cytoskeletal dynamics, intercellular junction stability, and vascular permeability, including pathways involving reversible protein phosphorylation regulating by the balance between serine/threonine kinase and phosphatase activities [10,11,12]. In addition, epigenetic mechanisms have emerged as important contributors to endothelial dysfunction with zinc-dependent histone deacetylases playing key roles in the regulation of inflammatory signaling and vascular barrier integrity during lung injury [13,14].

A broad range of natural and synthetic agents has been explored for their potential to modulate lung inflammation. Natural compounds such as mogroside V have demonstrated protective effects in LPS-induced ALI through the suppression of inflammatory signaling [15]. Lipoic acid exhibits antioxidative and anti-inflammatory activity across multiple respiratory disease models [16], while astaxanthin and resveratrol have been reported to attenuate oxidative stress and cytokine production in experimental lung injury [17,18]. Additionally, Vitamin D has been implicated in regulating pulmonary immune responses [19]. Beyond these natural agents, medicinal herbal extracts and marine-derived bioactives have shown activity against inflammatory pathways relevant to ALI [20,21]. Synthetic derivatives inspired by natural scaffolds, including Tanshinone-based analogs, have also demonstrated efficacy in preclinical ALI models [22]. Collectively, these studies highlight a growing interest in therapeutics that stabilize endothelial function, limit cytokine-driven injury, and promote the resolution of inflammation.

Chemically modified heparins represent a distinct class of molecules with relevance to inflammatory lung disease. Heparin and its derivatives can modulate inflammatory responses through multiple mechanisms, including interactions with cytokines, chemokines, proteases, damage-associated molecular patterns, and receptor-associated signaling complexes. These interactions are highly dependent on structural characteristics and sulfation patterns, which influence ligand binding and biological activity. Accordingly, non-anticoagulant heparins have attracted considerable interest as immunomodulatory agents capable of limiting inflammation while minimizing bleeding risk [23,24,25].

Among these derivatives, 2-O, 3-O-desulfated heparin (ODSH) is a chemically modified derivative of unfractionated heparin generated through the selective removal of the 2-O and 3-O sulfate groups. This modification substantially reduces anticoagulant activity while preserving the highly anionic glycosaminoglycan backbone and many of the non-anticoagulant biological properties of unfractionated heparin [25,26,27]. ODSH has an average molecular weight of approximately 11.7 ± 0.3 kDa [26] and retains strong electrostatic interactions with cationic proteins, including neutrophil elastase and cathepsin G, while maintaining several non-anticoagulant biological activities. Furthermore, ODSH has been shown to inhibit HMGB1 release by suppressing p300 acetyltransferase activity, thereby limiting HMGB1-dependent inflammatory signaling [27].

Thus, ODSH has been shown to inhibit neutrophil elastase [28], interfere with RAGE–ligand interactions [26], reduce HMGB1-mediated inflammation [27], and suppress the PF4/heparin complex formation associated with heparin-induced thrombocytopenia [29,30]. ODSH also mitigates thrombin-induced endothelial permeability [31], improves bacterial clearance and reduces lung injury in cystic fibrosis models [32], and has demonstrated safety in early-phase clinical evaluation in septic peritonitis [33] and acute myeloid leukemia (AML) [34,35]. These findings collectively suggest that ODSH modulates multiple inflammatory pathways relevant to ALI pathogenesis. A comprehensive discussion of ODSH biology, including its structure–function relationships, molecular targets, therapeutic applications, translational challenges, and outstanding research questions, has recently been summarized in our dedicated review [25].

Our group recently reported that ODSH protects against LPS-induced ALI in mice by reducing vascular leak, limiting neutrophil infiltration, and suppressing pro-inflammatory signaling while enhancing IL–10 expression [36,37]. While this work established ODSH as a possible candidate for ALI therapy, it did not address how ODSH influences endothelial junctional integrity, TLR4-dependent signaling, or the balance between pro-and anti-inflammatory mediators at the cellular level. The present study expands upon our earlier findings by integrating in vitro and in vivo approaches to define how ODSH modulates endothelial barrier function, cytokine regulation, and innate immune signaling. This work provides new insight into the anti-inflammatory and barrier-protective actions of ODSH in experimental ALI and establishes a foundation for future mechanistic and preclinical studies.

## 2. Materials and Methods

### 2.1. Cells, Chemicals and Reagents

Human lung microvascular endothelial cells (HLMVECs) were obtained from PromoCell (C-12281, Heidelberg, Germany) through the reseller MilliporeSigma (St. Louis, MO, USA). Unless indicated otherwise, reagents were obtained from Sigma–Aldrich (St. Louis, MO, USA). Lipopolysaccharide from *Escherichia coli* O111:B4 was used as the inflammatory stimulus. The Hanks’ balanced salt solution and phosphate-buffered saline solution were sourced from GIBCO (Invitrogen, Carlsbad, CA, USA). The 2x Laemmli sample buffer (Bio-Rad Laboratories, Hercules, CA, USA; Cat. No.1610737) was used for sample preparation prior to electrophoresis. Total protein content was quantified using the BCA protein assay kit (Pierce Chemical, Rockford, IL, USA) according to the manufacturer’s protocol. ODSH was supplied as a 50 mg/mL formulation by Cantex Pharmaceuticals (Weston, FL, USA).

### 2.2. MTT Assay for Assessment of Cellular Metabolic Activity Following ODSH Exposure

Cellular metabolic activity (commonly used as an indirect indicator of cell viability) was evaluated using the MTT assay [38,39,40] following 24 h exposure to ODSH. HLMVECs were seeded in 96-well plates at a density of 1 × 10^4^ cells per well and allowed to adhere overnight. Cells were then treated with vehicle control, hydrogen peroxide (200 µM; positive control), or increasing concentrations of ODSH (5, 25, 50, 100, and 500 µg/mL) for 24 h. After treatment, 10 µL MTT solution (stock: 5 mg/mL in PBS) was added in the medium (100 µL) and cells were incubated at 37 °C in a humidified CO_2_ incubator for 4 h. At the end of incubation, the supernatant was carefully aspirated, and 100 µL dimethyl sulfoxide (DMSO) was added to each well to solubilize the formazan crystals. The plate was kept in the dark at room temperature (23 ± 1 °C) for 30 min with gentle shaking. Absorbance was recorded at 570 nm using a microplate reader. Absorbance values were blank-corrected (cell–free wells) and data were normalized to vehicle-treated controls and expressed as relative metabolic activity. This approach is widely accepted to assess metabolic activity and cytotoxicity in experimental models of acute lung injury [41,42].

### 2.3. Transendothelial Electrical Resistance Measurement

The transendothelial electrical resistance (TER) of HLMVEC monolayers was assessed using an electric cell-substrate impedance sensing (ECIS Ζθ) impedance-based system (Applied BioPhysics, Troy, NY, USA) [31,43]. For TER measurements, 6 × 10^4^ cells per well were seeded onto 8W10E ECIS arrays and maintained in complete endothelial growth medium (EBM-2, Lonza, Walkersville, MD, USA) until a stable confluent monolayer was established. Confluent cultures were pretreated with either vehicle or 50 µg/mL ODSH for 40 min, which was followed by stimulation with 100 EU/mL LPS. TER was continuously recorded for the subsequent 24 h, normalized to baseline resistance values, and expressed as relative resistance over time.

### 2.4. Animal Experiments

All animal procedures were conducted in accordance with the Animal Welfare Act and were approved by the Institutional Animal Care and Use Committee (IACUC) of Augusta University under protocol 2011–0392 with the most recent approval issued on January 19, 2024. CD-1 mice (8–12 weeks of age, 20–25 g, 4 mice per group) were obtained from Charles River Laboratories (Wilmington, MA, USA). Animals were maintained in standard plastic cages with free access to food and water and housed under controlled environmental conditions, including ambient room temperature (23 ± 1 °C) and a 12 h light/dark cycle. Animals were randomly assigned to treatment groups (Control, ODSH, LPS, and ODSH + LPS) prior to experimental procedures. The sample size (*n* = 4 mice per group) was selected based on our previous experiments with the LPS-induced ALI model [36,37] and is consistent with similar mechanistic studies reported in the literature [44,45,46].

### 2.5. Animal Surgical Procedure

A murine model of acute lung injury was established using an intratracheal instillation of LPS under surgical exposure. Mice were anesthetized with an intraperitoneal mixture of ketamine (150 mg/kg) and acepromazine (15 mg/kg), after which the trachea was accessed through a midline cervical incision. A 20-gauge catheter was inserted directly into the trachea, and LPS (1 mg/kg in sterile PBS) was delivered into the lower airways. This approach provides a controlled deposition of LPS into the distal lung and minimizes the variability associated with aspiration-based delivery.

Fifteen minutes before LPS administration, animals received either vehicle or ODSH (50 mg/kg) through the internal jugular vein to ensure rapid systemic distribution. ODSH concentrations used in vitro and in vivo were selected based on our previously published studies demonstrating efficacy and tolerability in experimental ALI models [36,37]. Following treatment, mice recovered for 22 h. Anesthesia was then re-induced, and Evans blue dye albumin (EBDA; 30 mg/kg in 4% BSA) was administered via the same venous route. After a 120-min circulation period, animals were exsanguinated, the thoracic cavity was opened, and the pulmonary vasculature was flushed through the right ventricle with saline/EDTA to remove intravascular dye. Lungs were collected for downstream analyses, and bronchoalveolar lavage was performed by instilling 1 mL of 10% HBSS. The bronchoalveolar lavage fluid (BALF) was processed for protein quantification, cell counts, and cytokine measurements. Lung tissues were stored at −80 °C for RT-qPCR and Western blot analyses.

A separate cohort of animals was used for the EBDA permeability endpoint, as all measurements could not be reliably obtained from a single group of mice. One cohort was dedicated exclusively to lung permeability assessment. A second cohort was used for wet–to–dry lung weight ratio measurements, BALF analyses (total protein, cell counts, IL-6 and TNF-α quantification), and molecular studies (RT-qPCR and Western blotting).

### 2.6. Lung Permeability Measurement Using Evans Blue Dye Albumin

Vascular permeability was quantified using a widely accepted fluorescent azo dye, Evans blue dye albumin measurement as we have previously described [36]. Briefly, EBDA (30 mg/kg) was administered via the right internal jugular vein two hours before the terminal procedure. After euthanasia and vascular flushing, the blood-free left lung was excised, weighed, and frozen at −20 °C. Dye was extracted by homogenizing the tissue in formamide and incubating the homogenate for 18 h at 60 °C. Samples were centrifuged at 5000× *g* for 30 min, and absorbance of the supernatant was measured at 620 nm and 750 nm to correct for background signal. The EBDA content was calculated from a standard curve and normalized to lung tissue weight to determine relative permeability.

### 2.7. Lung Weight to Dry Ratio

Lung edema was quantified by determining the lung wet-to-dry weight ratio. After terminal anesthesia and thoracotomy, lungs were perfused via the right ventricle with ice-cold PBS to remove intravascular blood. The right middle lobe was excised, gently blotted to remove surface fluid, and immediately weighed to obtain wet weight. Samples were then dried in an oven at 60 °C for 48–72 h until constant weight was achieved and reweighed to determine dry weight. The wet-to-dry lung ratio was calculated as wet weight divided by dry weight for each animal lung.

### 2.8. Protein Estimation and Cell Count from the BALF

Bronchoalveolar lavage fluid was processed immediately after collecting it to separate cellular and acellular components. The lavage was first centrifuged at 500× *g* for 15 min at 4 °C to pellet cells. The supernatant was transferred to a fresh tube and subjected to a second centrifugation at 2500× *g* for 10 min at 4 °C to obtain clarified BALF for total protein measurement. The initial cell pellet was resuspended in ACK lysing buffer and incubated for 15 min with gentle mixing to remove residual erythrocytes. Samples were then centrifuged again at 500× *g* for 15 min at 4 °C. The resulting pellet was fixed in 3.7% formalin, and total cell numbers were determined using a hemocytometer under light microscopy. Total protein concentration in BALF was quantified using the BCA protein assay kit (Pierce Chemical, Rockford, IL, USA) according to the manufacturer’s protocol and expressed as µg/mL.

### 2.9. Quantification of IL-6 and TNF-α in BALF

Clarified BALF was prepared by sequential centrifugation at 500× *g* for 15 min at 4 °C, which was followed by a second spin under identical conditions to remove residual debris. Concentrations of IL-6 and TNF-α in the supernatant were measured using the ELISA MAX™ Deluxe Set (BioLegend, San Diego, CA, USA) according to the manufacturer’s instructions. Absorbance was recorded at 450 nm, and background correction was performed by subtracting the signal at 570 nm from the primary reading. Cytokine levels were calculated from standard curves and expressed as pg/mL.

### 2.10. Quantitative Real-Time PCR Analysis (RT-qPCR)

Total RNA was extracted from HLMVECs and mouse lung homogenates using TRIzol reagent (Invitrogen, Carlsbad, CA, USA) following the manufacturer’s recommended protocol. RNA concentration and purity were assessed using a NanoDrop spectrophotometer. For each sample, 2 µg of total RNA was reverse–transcribed into cDNA using the High-Capacity cDNA Reverse Transcription Kit (Applied Biosystems).

RT-qPCR reactions were prepared in a 20 µL volume in MicroAmp™ Fast Optical 96-well plates and contained 5 µL of cDNA (100 ng), 10 µL of PowerUp™ SYBR™ Green Master Mix (2×), 3 µL nuclease–free water, and 2 µL of gene–specific primers (1 µL each of forward and reverse primers at 10 µM). Amplification was performed on the StepOnePlus™ Real-Time PCR System (Applied Biosystems, Foster City, CA, USA).

Gene expression was quantified for IL-1β, TNF-α, IL-6, RELA, NOS2, MAPK14, MAPK1, ICAM1, CCL2, IL-10, CDH5, OCLN, CLDN5, TJP1, TLR4, and MYD88 with HPRT1 serving as the reference gene. Relative mRNA levels were calculated using the ΔΔCt method with expression changes reported as 2^−ΔΔCt^. The primer sequences used for both HLMVEC and mouse lung analyses are provided in Supplementary Tables S1 and S2.

### 2.11. Protein Extraction and Immunoblotting

Protein lysates were prepared from mouse lung tissue and HLMVECs to evaluate the expression of inflammatory and signaling proteins. Lung tissue (that had been stored at −80 °C prior to processing) was minced on ice and homogenized in lysis buffer containing 2× Laemmli sample buffer with protease inhibitors, phosphatase inhibitors, and β-mercaptoethanol. HLMVECs were washed with cold PBS and lysed directly on the culture dish using the same inhibitors-supplemented buffer. Homogenates and cell lysates were incubated on ice and clarified by centrifugation to obtain total protein extracts suitable for Western blotting.

Protein samples were separated by SDS–PAGE and transferred onto PVDF membranes. Membranes were blocked and probed with primary antibodies targeting NF-κB p65 (4764S, monoclonal, 1:1000), MyD88 (4283S, monoclonal, 1:1000), and TNF-α (3707S, polyclonal, 1:1000), which were all obtained from Cell Signaling Technology (Danvers, MA, USA). The housekeeping protein β-actin (HRP-66009, HRP-conjugated monoclonal, 1:1000; Proteintech Group, Rosemont, IL, USA) served as the loading control.

After incubation with HRP-conjugated secondary antibodies, immunoreactive bands were visualized using an ECL detection kit. Band intensities were quantified and normalized to β-actin to determine the relative protein expression across experimental groups. Densitometric measurements were performed using ImageJ software (Research Services Branch, NIH, Bethesda, MD, USA).

### 2.12. Statistical Analysis

Data are presented as mean ± SEM unless otherwise indicated. Cell culture experiments represent 4–6 independent biological replicates, whereas animal experiments were performed using 4 mice per group. Because the sample size was small and normal distribution could not be assumed, nonparametric tests were used. Multi-group comparisons were analyzed using the Kruskal–Wallis test followed by Dunn’s post hoc test, while two-group comparisons were assessed with the Mann–Whitney test. A *p*-value of ≤0.05 was considered statistically significant. All statistical analyses and graph generation were carried out using GraphPad Prism software (Version 10.5; San Diego, CA, USA).

## 3. Results

### 3.1. ODSH Does Not Significantly Affect HLMVEC Metabolic Activity at Therapeutically Relevant Concentrations

**Figure 1:**
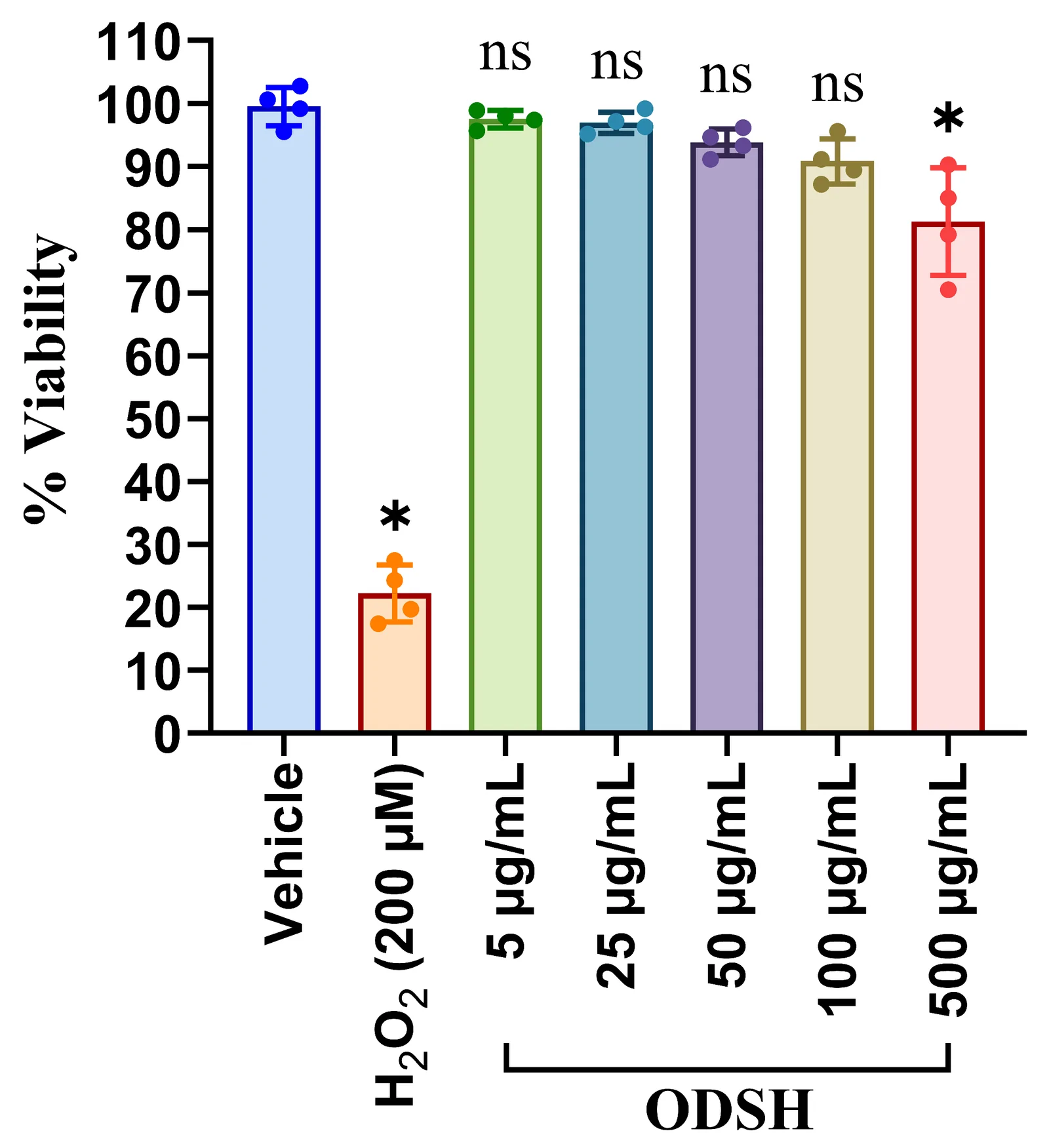
Effects of ODSH on HLMVEC metabolic activity. Cells were treated for 24 h with vehicle, H_2_O_2_ (200 µM), or various concentrations of ODSH (5, 25, 50, 100, and 500 µg/mL). Vehicle-treated cells showed full metabolic activity, whereas H_2_O_2_, used as a positive control for oxidative injury, markedly reduced viability. ODSH at 5–100 µg/mL did not significantly affect cellular metabolic activity and maintained viability comparable to vehicle-treated controls, while 500 µg/mL produced a modest, dose-dependent decrease. Data expressed as mean ± SD (*n* = 4). * Values significant at *p* < 0.05 as against the Vehicle; ns = not significant as against the Vehicle.

The treatment of HLMVECs with ODSH for 24 h revealed a clear separation between non-toxic and mildly cytotoxic dose ranges. Vehicle-treated cells maintained full metabolic activity, whereas exposure to 200 µM hydrogen peroxide produced a pronounced reduction in viability, confirming assay sensitivity. ODSH at concentrations of 5–100 µg/mL did not significantly alter cellular metabolic activity relative to vehicle-treated controls. In contrast, treatment with 500 µg/mL ODSH resulted in a modest but reproducible, dose-dependent decrease in metabolic activity. It is worth noting that the therapeutic concentrations of ODSH in humans ranged from ~20 ug/mL up to 75 ug/mL [34,35], which is consistent with our observations. These findings demonstrate that ODSH is primarily non-toxic to HLMVECs across a broad concentration range with only the highest (apparently not a therapeutic) dose producing a measurable reduction in viability (Figure 1).

### 3.2. ODSH Significantly Attenuates the LPS-Mediated Decrease in Transendothelial Electrical Resistance

**Figure 2:**
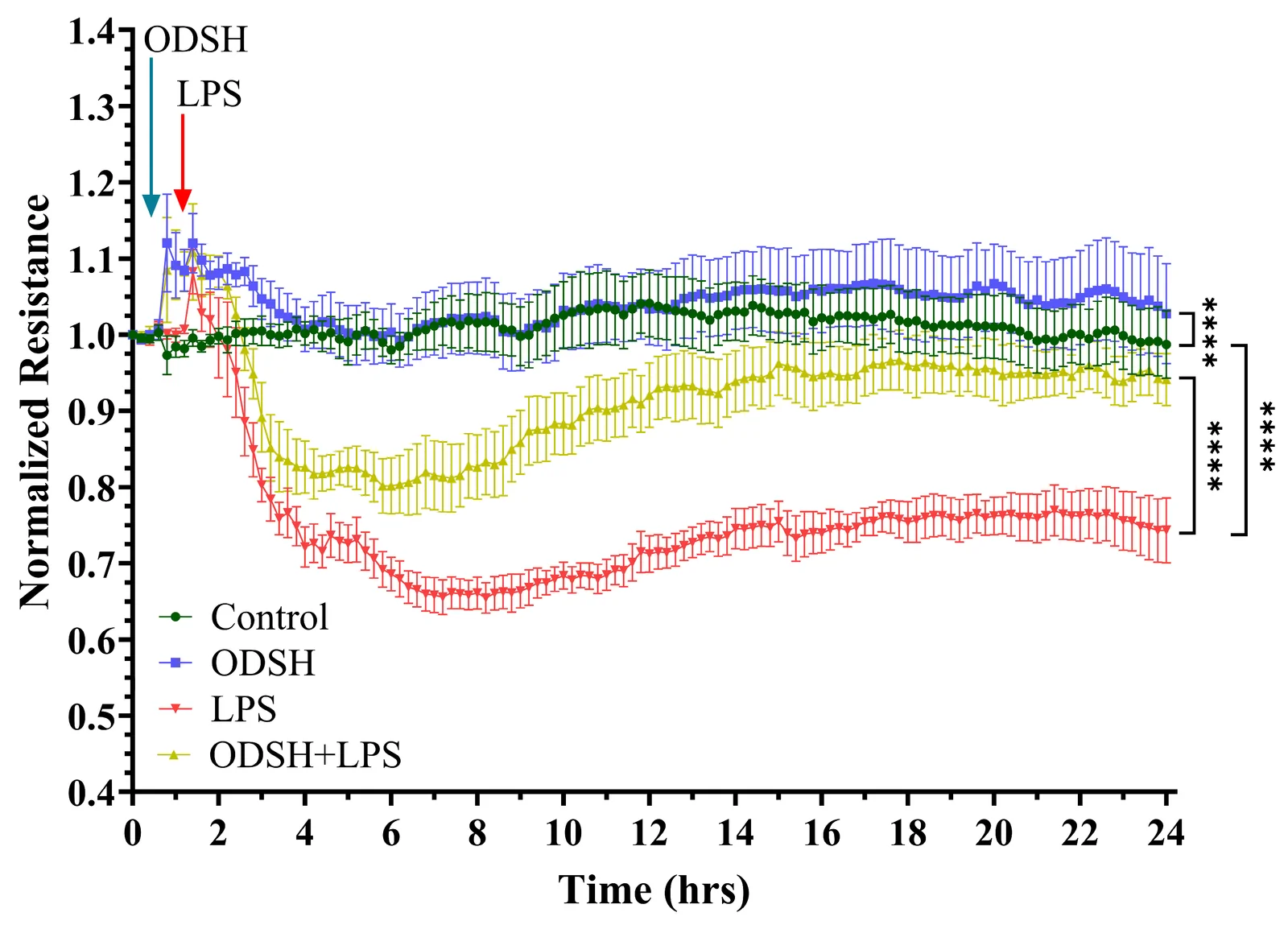
Effect of ODSH on LPS-induced endothelial barrier disruption. Pretreatment with 50 μg/mL ODSH for 40 min attenuated the decrease in transendothelial electrical resistance (TER) caused by 100 EU/mL LPS in human lung microvascular endothelial cell monolayers cultured on ECIS arrays. The timing of effector addition is indicated by arrows. TER measurements were normalized to baseline resistance and expressed as Mean ± SEM (*n* = 6). **** *p* < 0.0001.

The stimulation of confluent HLMVEC monolayers with 100 EU/mL LPS produced a rapid and sustained decline in transendothelial electrical resistance, which is consistent with marked barrier disruption. Pretreatment with 50 µg/mL ODSH for 40 min alone modestly but significantly increased basal TER and significantly attenuated the LPS-mediated decrease in TER throughout the 24 h monitoring period, indicating a partial preservation of endothelial barrier integrity (Figure 2). These findings demonstrate that ODSH mitigated the LPS-induced decline in endothelial barrier function.

### 3.3. ODSH Pretreatment Efficiently Reduces LPS-Induced Capillary Leak and Edema in the Murine Model of ALI

**Figure 3:**
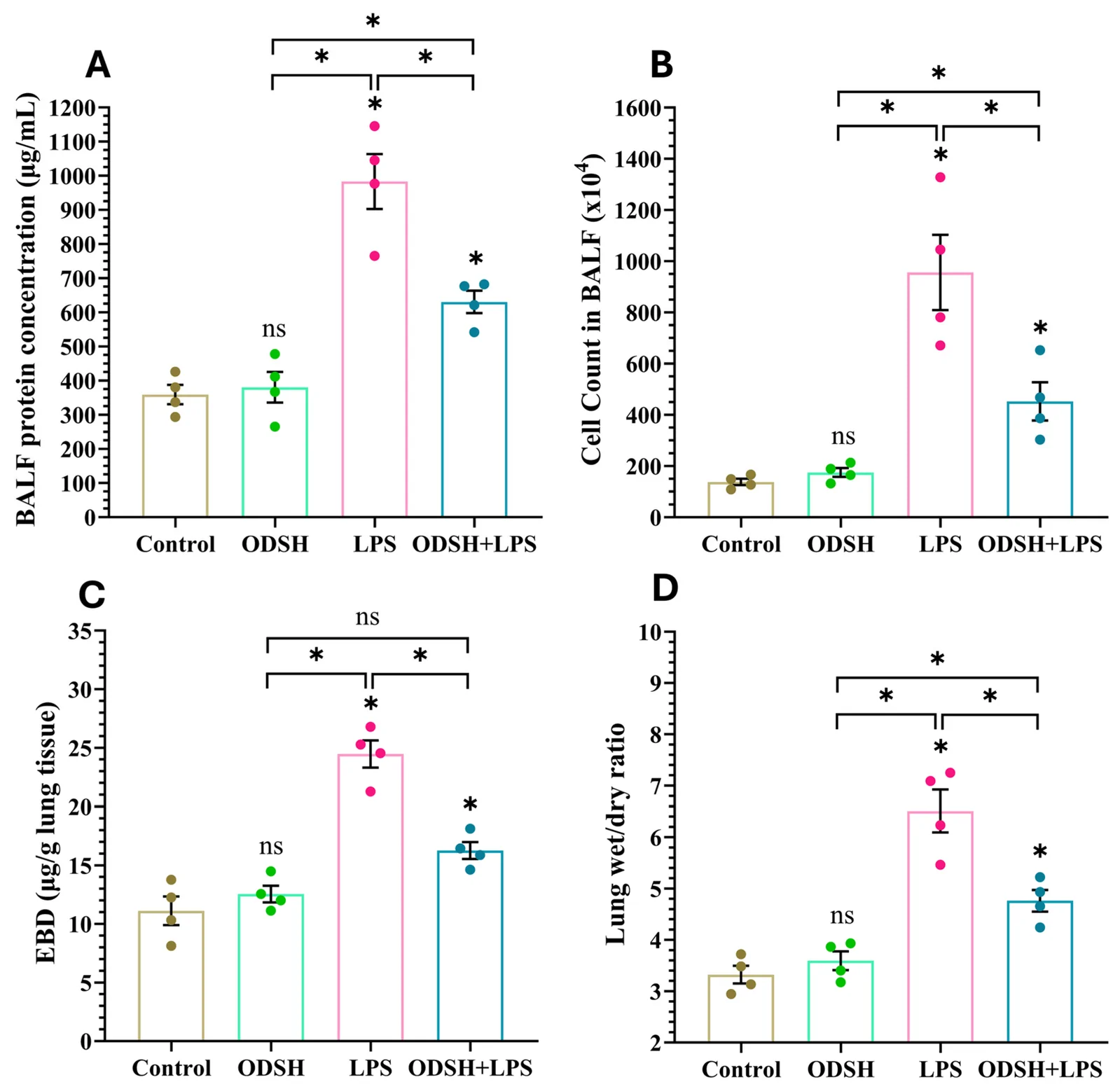
Bronchoalveolar lavage fluid (BALF) protein levels, inflammatory cell counts, and Evans blue dye albumin (EBDA) leakage and wet/dry ratio measurement in lung tissue. (**A**) Pretreatment with ODSH (50 mg/kg, administered via the IJV 15 min before IT LPS) lowered total protein accumulation in the BALF of mice exposed to LPS (1 mg/kg). (**B**) ODSH also reduced the number of white blood cells recovered in BALF following LPS challenge compared with untreated LPS-exposed mice. (**C**) EBDA (30 mg/kg) was administered through the IJV 2 h prior to the end of the experiment. LPS markedly increased EBDA extravasation from the vasculature into lung tissue, whereas ODSH pretreatment substantially limited this leakage. (**D**) LPS increased lung wet/dry weight ratio, reflecting pulmonary edema. ODSH pretreatment significantly reduced the LPS-induced rise in W/D ratio, indicating an attenuation of lung fluid accumulation and improved barrier integrity. Data are shown as mean ± SEM (*n* = 4 mice). ns = not significant, * *p* < 0.05. Comparisons were made against the control group unless otherwise indicated.

To determine whether ODSH mitigates LPS-driven ALI in mice, we evaluated multiple indices of lung permeability and inflammation. LPS challenge markedly increased protein accumulation in BALF, whereas mice pretreated with ODSH exhibited a substantial reduction in BALF protein levels (Figure 3A). A similar protective effect was observed for inflammatory cell recruitment: ODSH pretreatment significantly decreased BALF leukocyte counts compared with LPS alone (Figure 3B). Consistent with these findings, LPS exposure led to a pronounced extravasation of Evans blue dye albumin into lung tissue, reflecting increased microvascular permeability. ODSH administration markedly reduced this EBDA leakage, which is consistent with improved vascular barrier function (Figure 3C). We further assessed pulmonary edema by measuring the wet-to-dry lung weight ratio. LPS significantly elevated the W/D ratio, whereas ODSH pretreatment attenuated this increase, demonstrating reduced fluid accumulation and improved barrier function (Figure 3D).

### 3.4. ODSH Mitigates Increase in IL-6 and TNF-α Expression in LPS-Induced ALI Mice Model

**Figure 4:**
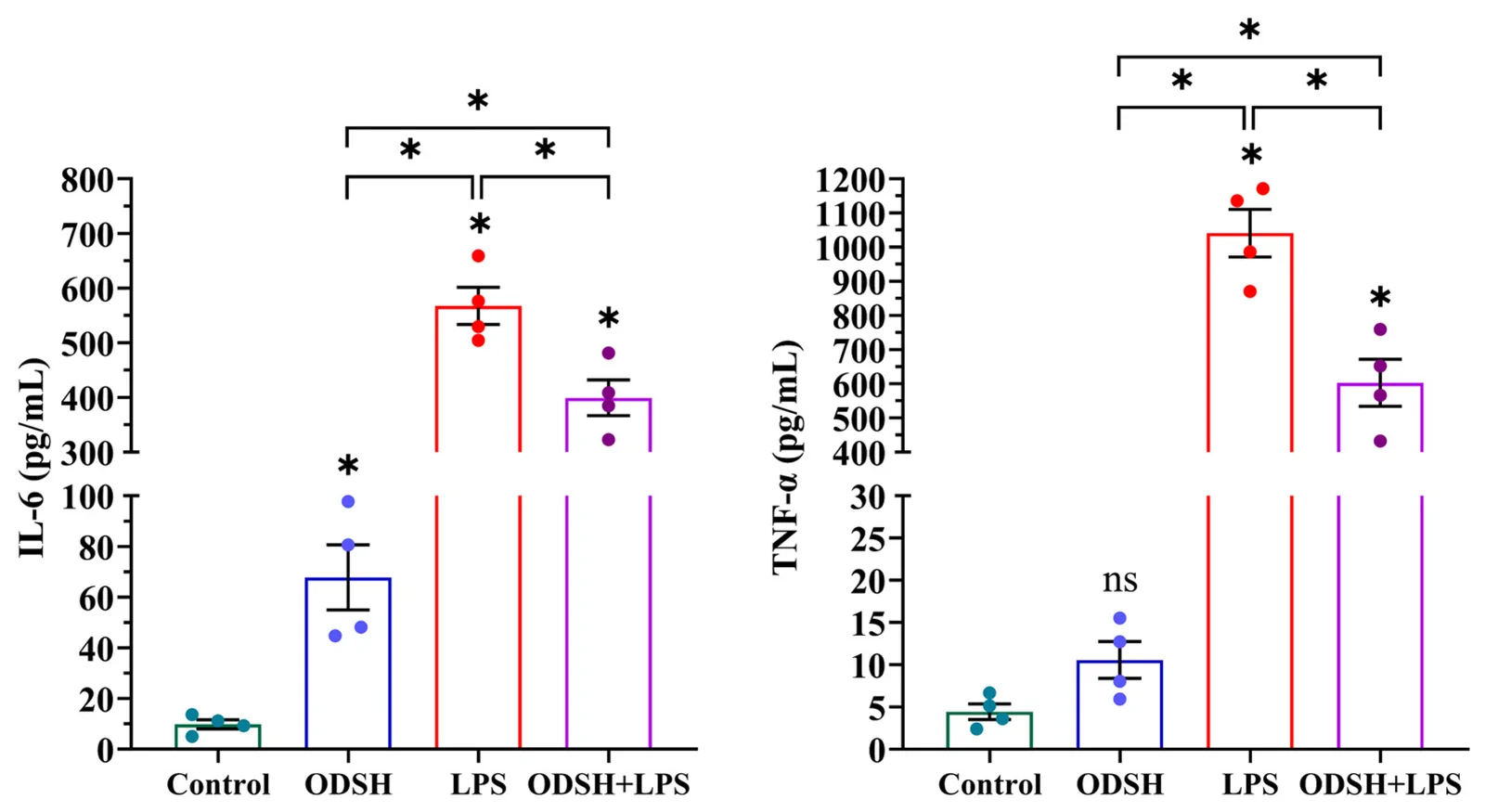
Effects of LPS on IL-6 and TNF-α levels in mouse BALF and the modulatory action of ODSH. ELISA analysis showed that both cytokines were elevated in the BALF of mice exposed to LPS alone. Pretreatment with ODSH (50 mg/kg body weight, administered via the IJV 15 min prior to IT LPS) substantially reduced the LPS-induced rise in IL-6 and TNF-α, demonstrating attenuation of the inflammatory response in the ODSH/LPS group. Mean ± SEM (*n* = 4). ns: not significant, * *p* < 0.05. Comparisons were made against the control group unless otherwise indicated.

To verify if ODSH modulates the inflammatory cytokine response associated with LPS-induced lung injury, we quantified IL-6 and TNF-α levels in BALF. LPS challenge resulted in a pronounced increase in both cytokines, which is consistent with a strong activation of inflammatory cascade. Pretreatment with ODSH markedly reduced these elevations, leading to significantly lower IL-6 and TNF-α concentrations compared with the LPS-only group. This attenuation indicates that ODSH effectively limits pro-inflammatory responses triggered by LPS in the murine ALI model (Figure 4).

### 3.5. ODSH Inhibits LPS-Induced Expression of Pro-Inflammatory Genes in HLMVECs and Mouse Lung Tissue

**Figure 5:**
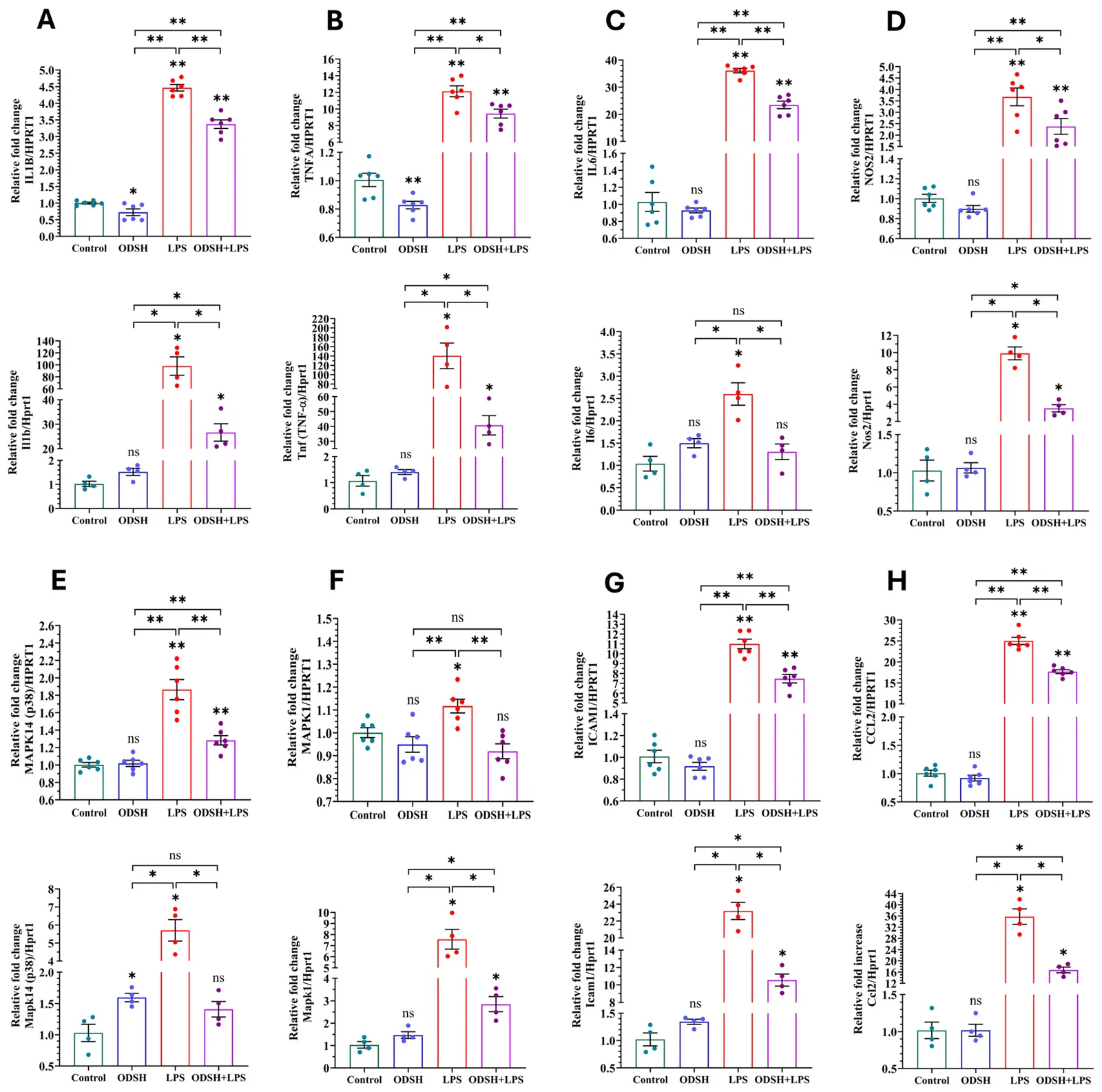
Relative mRNA expression of pro-inflammatory genes: (**A**) IL-1B, (**B**) TNFA (TNF-α), (**C**) IL-6, (**D**) NOS2 (iNOS), (**E**) MAPK14 (p38), (**F**) MAPK1, (**G**) ICAM1 and (**H**) CCL2. Quantitative analysis of mRNA levels was performed in HLMVECs and mouse lung tissue with expression normalized to HPRT1. While exposure to LPS alone led to a marked upregulation of the inflammatory markers, the co-administration of ODSH significantly reduced their expression, indicating a suppressive effect on pro-inflammatory signaling. Data are shown as mean ± SEM (*n* = 6 for HLMVECs, *n* = 4 for Mice). ns = not significant, * *p* < 0.05, ** *p* < 0.01. Comparisons were made against the control group unless otherwise indicated.

LPS stimulation produced a vigorous transcriptional activation of multiple pro-inflammatory mediators in both HLMVECs and mouse lung tissue, which is reflected by marked increases in IL-1β, TNF-α, IL-6, NOS2, MAPK14 (p38), MAPK1, ICAM1, and CCL2 mRNA levels. ODSH co-treatment substantially restricted these LPS-driven elevations across all measured targets, demonstrating a broad suppressive effect on inflammatory gene expression. The coordinated reduction in inflammatory transcripts suggests that ODSH modulates pathways involved in LPS-induced inflammatory responses. These findings support a consistent inhibitory action of ODSH on LPS-induced transcriptional activation in endothelial cells and lung tissue (Figure 5).

### 3.6. ODSH Enhances LPS-Induced Anti-Inflammatory Signaling at Transcriptional Level

**Figure 6:**
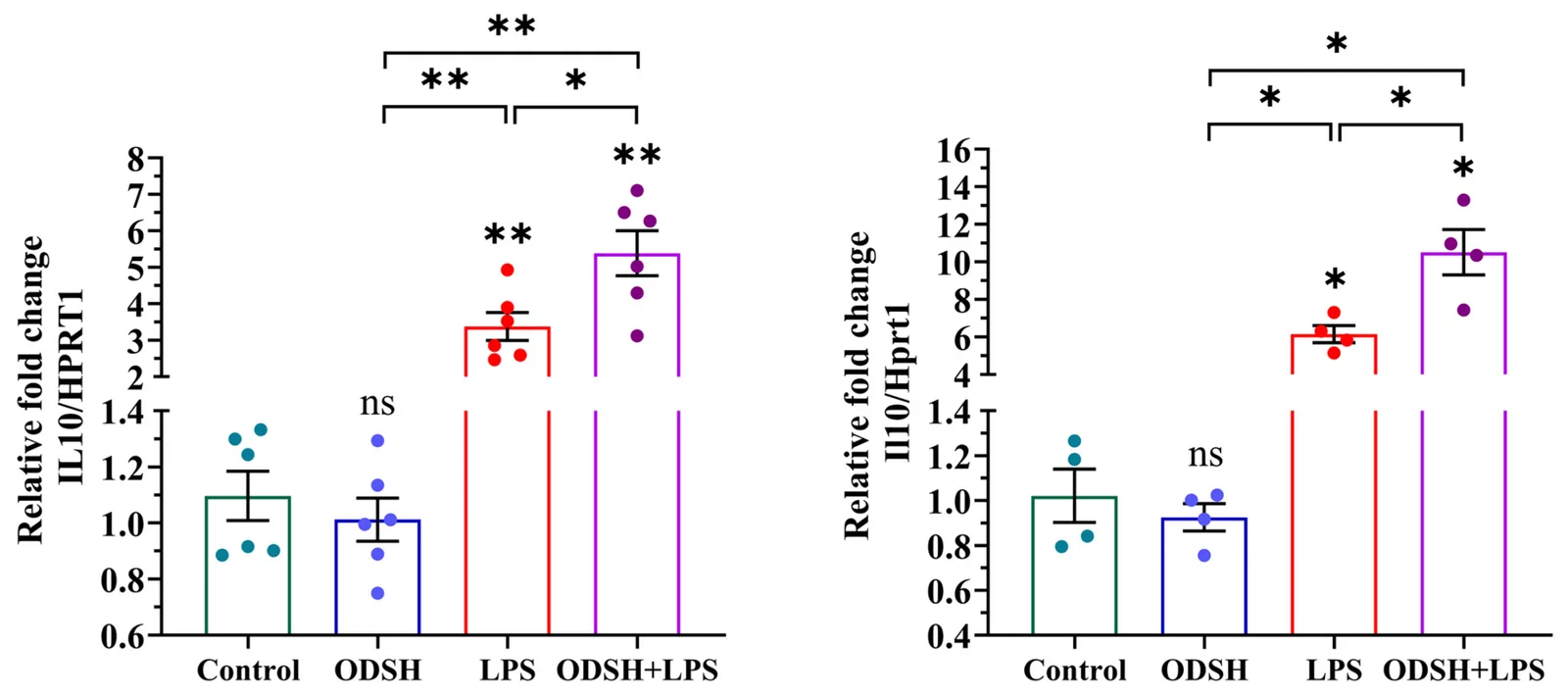
Relative mRNA expression of anti-inflammatory gene: IL-10 in HLMVECs and mouse lungs. Expression normalized to HPRT1. LPS exposure led to a marked upregulation of IL-10 expression, while co-administration of ODSH further enhanced IL-10 expression, indicating a protective effect on anti-inflammatory signaling. Data are shown as mean ± SEM (*n* = 6 for HLMVECs, *n* = 4 for Mice). ns = not significant, * *p* < 0.05, ** *p* < 0.01. Comparisons were made against the control group unless otherwise indicated.

Consistent with data from the literature [47,48], LPS stimulation increases the expression of key anti-inflammatory cytokine IL-10 in HLMVECs and mouse lung tissue, thus providing negative anti-inflammatory feedback. ODSH co-treatment further increases the levels of IL-10 transcript beyond the level observed with LPS alone, reflecting a further activation of compensatory regulatory pathways. These findings suggest that ODSH may enhance compensatory anti-inflammatory responses during LPS challenge (Figure 6).

### 3.7. ODSH Treatment Was Found Effective in Endothelial Junction Genes Against LPS Challenge

**Figure 7:**
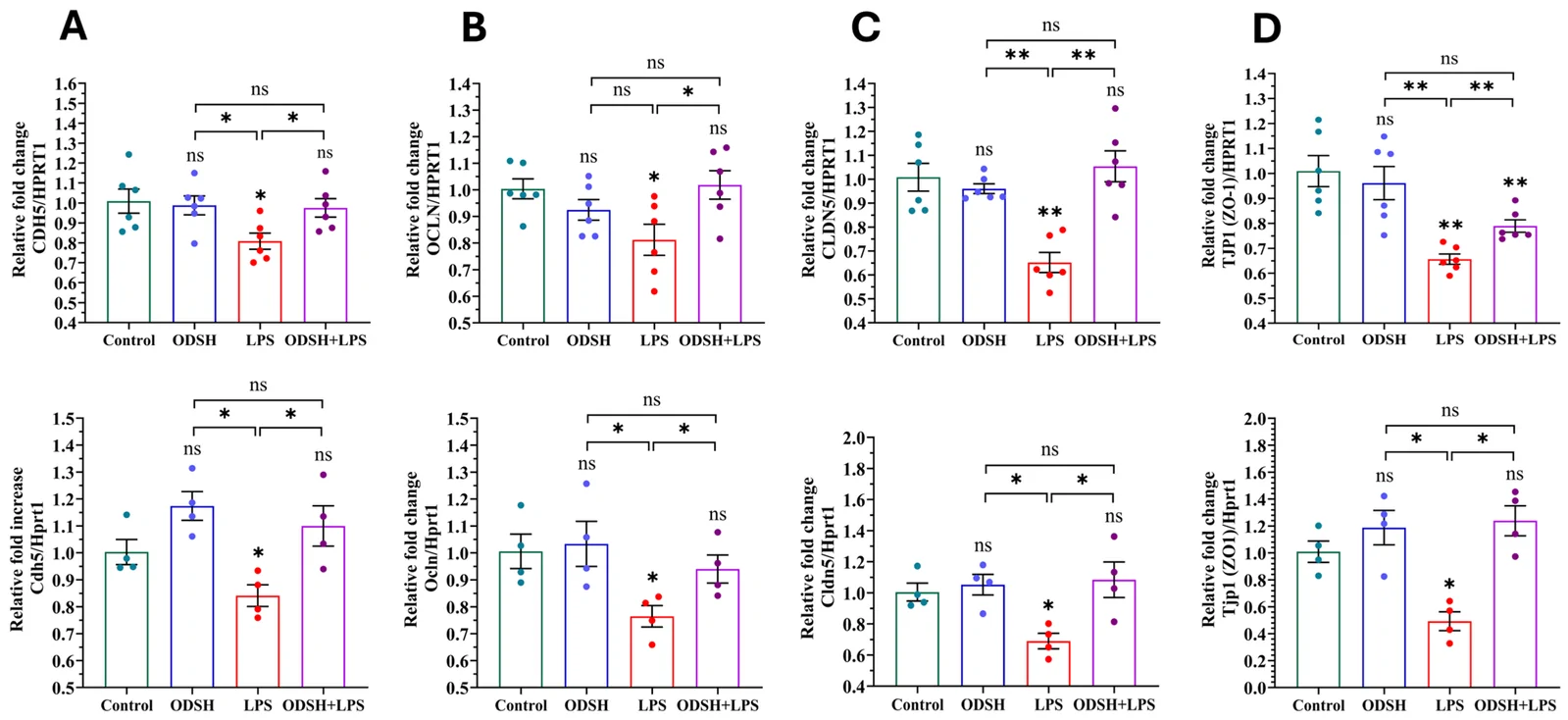
Impact of ODSH on relative mRNA expression of endothelial junctional genes: (**A**) CDH5, (**B**) OCLN, (**C**) CLDN5, and (**D**) TJP1 (ZO-1). LPS treatment in HLMVECs and mouse lung tissue resulted in a marked downregulation of all four junctional transcripts, indicating a disruption of endothelial barrier integrity. The co-administration of ODSH mitigated these LPS-induced reductions, restoring gene expression toward baseline, supporting the preservation of endothelial barrier-associated transcriptional programs. Data are shown as mean ± SEM (*n* = 6 for HLMVECs, *n* = 4 for Mice). ns = not significant, * *p* < 0.05, ** *p* < 0.01. Comparisons were made against the control group unless otherwise indicated.

The disruption of endothelial barrier structure was evident in both HLMVECs and mouse lung tissue after LPS exposure, as reflected by the reduced expression of key junctional integrity genes, including CDH5 (VE–cadherin), OCLN, CLDN5, and TJP1 (ZO-1). The co-administration of ODSH counteracted these LPS-induced declines, restoring transcript levels toward baseline across all four markers. The improvement in adherens and tight junction gene expression suggests that ODSH restores the expression of junction-associated genes that are important for endothelial barrier maintenance (Figure 7), complementing its permeability protective effects observed in vivo and in vitro.

### 3.8. ODSH Attenuates Expression of Pro-Inflammatory Genes Involved in LPS Signaling

**Figure 8:**
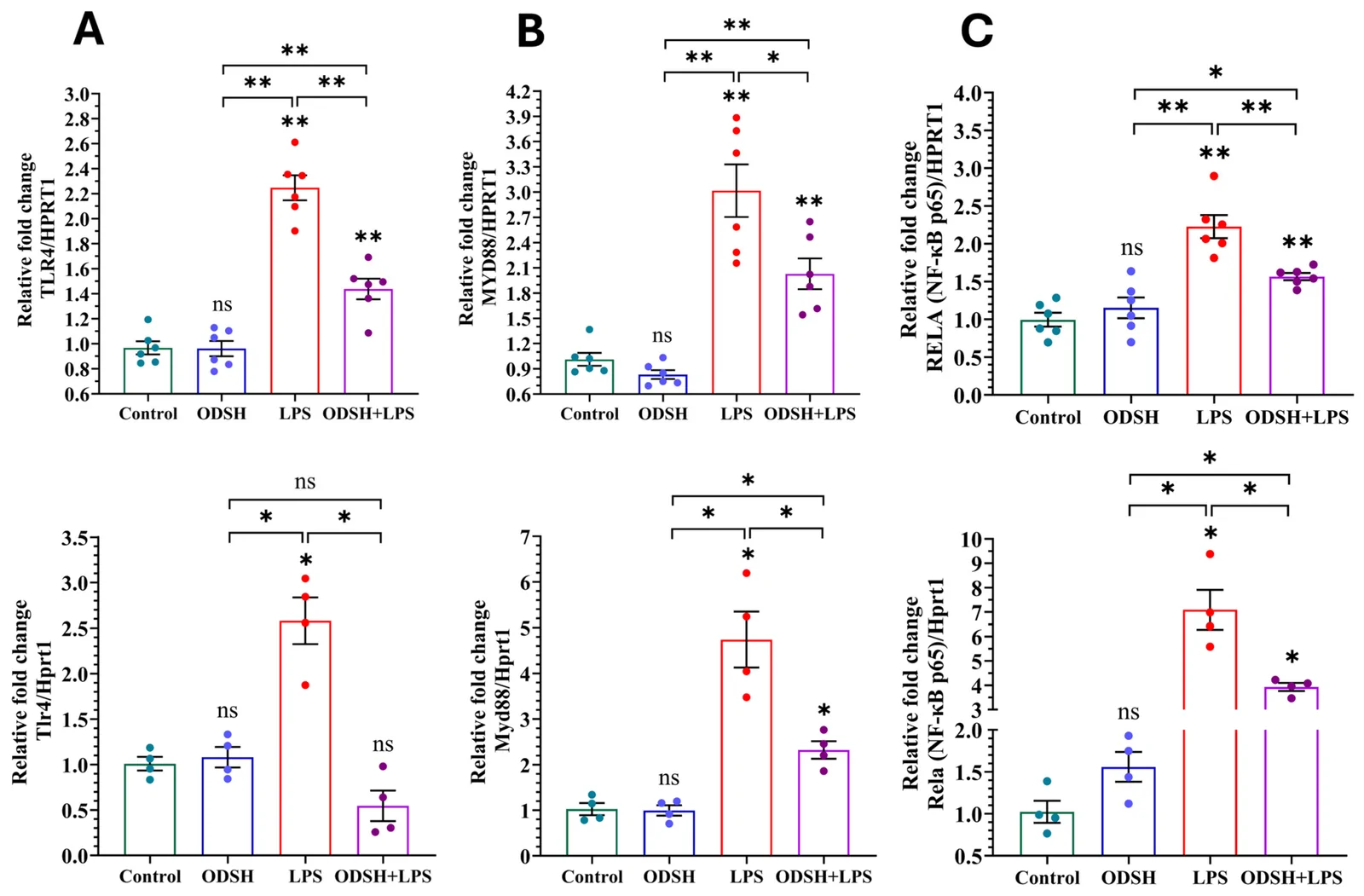
ODSH mitigates LPS-induced upregulation of pro-inflammatory genes. Relative mRNA expression levels of (**A**) TLR4, (**B**) MYD88, and (**C**) RELA (NF-κB) were quantified in HLMVECs and mouse lung following LPS stimulation and ODSH treatment. LPS markedly increased TLR4, MYD88, and RELA levels. ODSH treatment significantly reversed these LPS-induced alterations. Data are shown as mean ± SEM (*n* = 6 for HLMVECs, *n* = 4 for Mice). ns = not significant, * *p* < 0.05, ** *p* < 0.01. Comparisons were made against the control group unless otherwise indicated.

LPS stimulation triggered a strong transcriptional activation of key signaling components with marked increases in TLR4, MYD88, and RELA (NF-κB) expression in both HLMVECs and mouse lung tissue. These elevations reflect the activation of the canonical TLR4–MyD88–NF-κB axis that drives early inflammatory responses. The co-administration of ODSH effectively countered these LPS-induced changes, restoring transcript levels of all three genes toward baseline. The reversal of TLR4, MYD88, and RELA upregulation suggests that ODSH modulates the expression of key components of the TLR4–MyD88–NF-κB signaling pathway (Figure 8). 

### 3.9. ODSH Mitigates the LPS-Induced Upregulation of Pro-Inflammatory Protein Expression in HLMVECs

**Figure 9:**
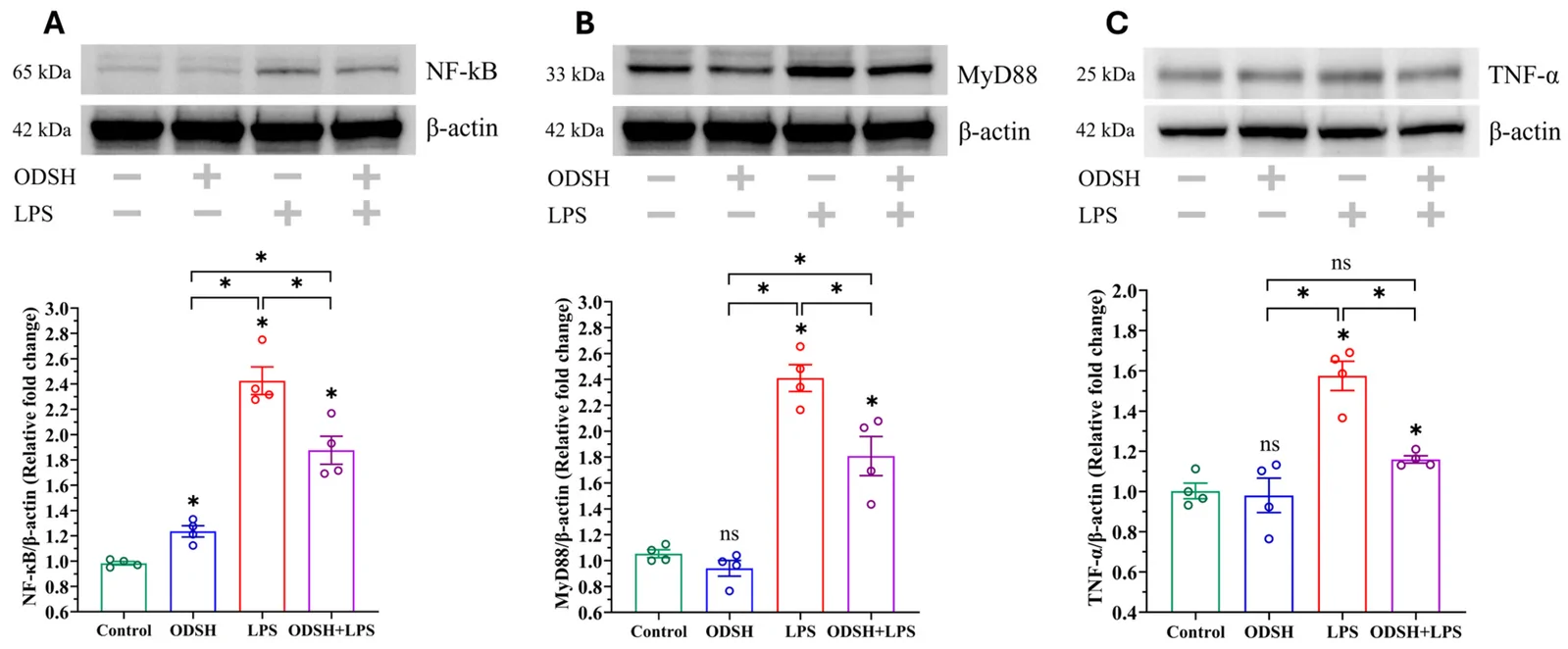
Effects of LPS on NF-κB, MyD88, and TNF-α protein expression in HLMVECs. Immunoblot analysis demonstrated that LPS stimulation markedly increased the expression of (**A**) NF-κB, (**B**) MyD88, and (**C**) the pro-inflammatory cytokine TNF-α compared with control cells. Please refer to Supplementary Figure S1 for original Western blot images. Co-treatment with ODSH attenuated these LPS-induced elevations, which is consistent with the modulation of inflammatory signaling pathways in endothelial cells. Mean ± SEM (*n* = 4). ns: not significant, * *p* < 0.05. Comparisons were made against the control group unless otherwise indicated.

Consistent with the transcriptional changes described in Section 3.8 and Section 3.5, immunoblot analysis confirmed that LPS markedly increased the protein expression of NF-κB, MyD88, and the pro-inflammatory cytokine TNF-α in HLMVECs. These protein-level elevations are consistent with enhanced inflammatory signaling in response to LPS challenge. ODSH co-treatment substantially reduced the abundance of all three proteins, validating the suppressive effects observed at the mRNA level and demonstrating that ODSH attenuates LPS-associated increases in inflammatory protein expression. The attenuation of NF-κB, MyD88, and TNF-α protein expression further supports ODSH’s role in dampening proximal and downstream inflammatory responses in endothelial cells (Figure 9).

### 3.10. ODSH Mitigates the LPS-Induced Upregulation of Pro-Inflammatory Proteins in Mice

**Figure 10:**
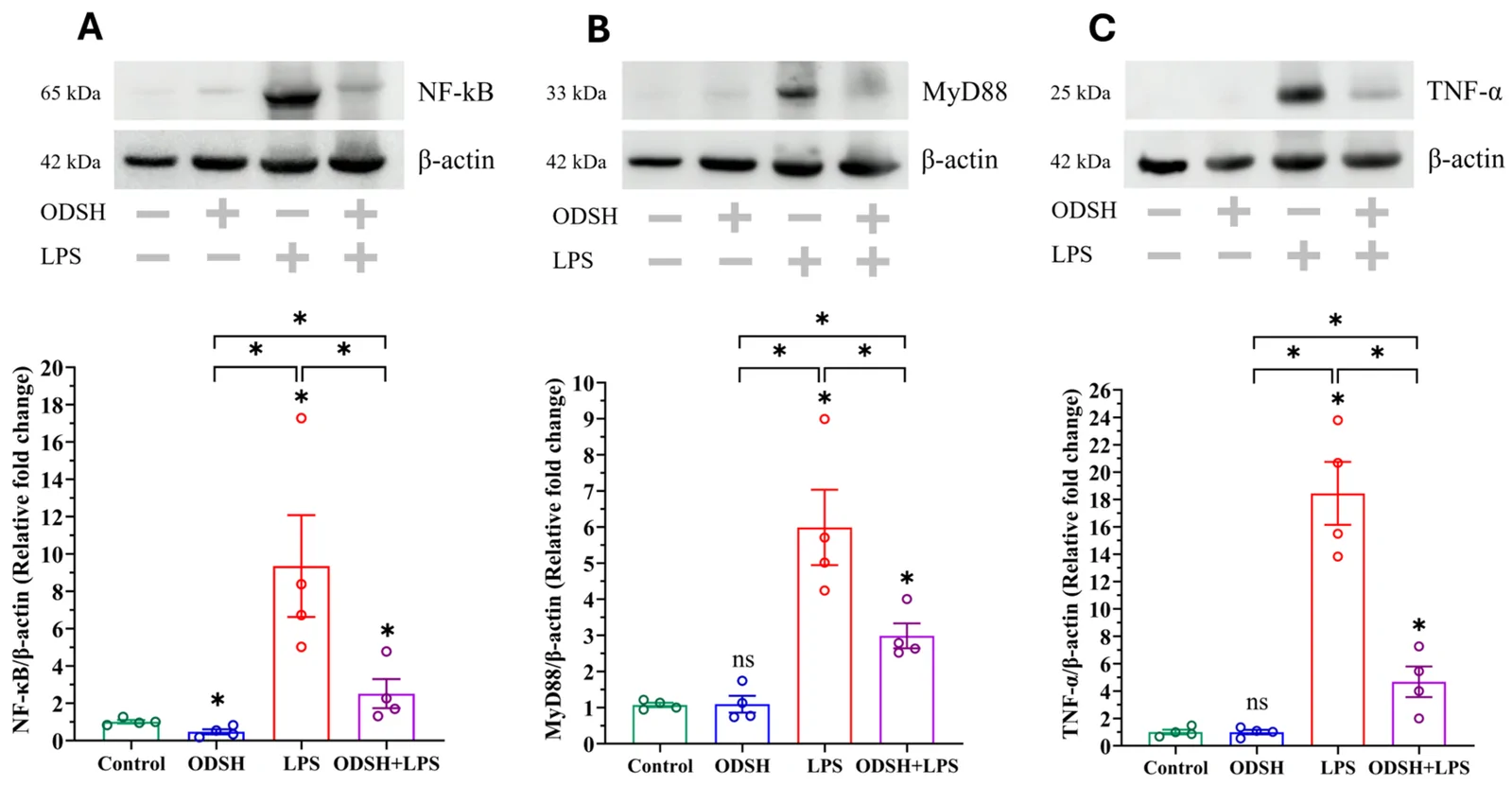
Effects of LPS on NF-κB, MyD88, and TNF-α protein expression in mouse lung tissue. LPS challenge in vivo resulted in significant upregulation of (**A**) NF-κB, (**B**) MyD88, and (**C**) TNF-α at 24 h, reflecting activation of inflammatory signaling pathways during acute lung injury. Please refer to Supplementary Figure S2 for original Western blot images. ODSH administration significantly attenuated these increases, demonstrating its protective effect on pulmonary inflammatory responses. Mean ± SEM (*n* = 4). ns: not significant, * *p* < 0.05. Comparisons were made against the control group unless otherwise indicated.

To extend our in vitro observations, we next examined whether ODSH similarly modulates LPS-driven inflammatory signaling in vivo. An immunoblot analysis of mouse lung lysates showed pronounced increases in NF-κB, MyD88, and TNF-α protein levels following LPS challenge, which is consistent with enhanced inflammatory signaling in the injured lung. ODSH pretreatment markedly attenuated the expression of all three proteins, restoring their levels toward those observed in control lungs. These findings further validate the transcriptional changes described in Section 3.8 (Section 3.5 for TNF-α) and support the conclusion that ODSH modulates inflammatory responses at both transcriptional and protein expression levels in vivo (Figure 10).

## 4. Discussion

The present study expands our understanding of the protective actions of ODSH in LPS-induced acute lung injury by demonstrating its coordinated effects on endothelial viability, inflammatory signaling, and vascular barrier integrity in both endothelial cells and mouse lung tissue. Consistent with our earlier findings [36], ODSH again showed a strong capacity to limit LPS-driven vascular leak and inflammatory cell recruitment, and the current work provides additional mechanistic insight into how these protective effects are achieved at the molecular and cellular levels.

Initial viability studies confirmed that ODSH is well tolerated by endothelial cells across a broad concentration range. Doses between 5 and 100 µg/mL did not affect HLMVEC metabolic activity, while only the highest concentration produced a modest reduction in viability. These findings discriminate clearly a safety concern of ODSH and support the use of physiologically relevant doses in subsequent mechanistic experiments. In parallel, ODSH preserved endothelial barrier function in vitro, as evidenced by its ability to mitigate the LPS-mediated decline in transendothelial electrical resistance. The stabilization of TER indicates that ODSH preserves endothelial barrier function under inflammatory stress [31,49].

The in vivo findings further support the barrier-protective actions of ODSH. In our previous study, we have shown that LPS challenge produced the anticipated increases in BALF protein content, inflammatory cell accumulation, EBDA extravasation, and lung edema [36], which are hallmarks of microvascular injury in ALI [50,51,52]. ODSH pretreatment significantly attenuated each of these parameters, indicating reduced permeability, diminished inflammatory infiltration, and improved fluid homeostasis. These findings are consistent with our previous observations demonstrating protective effects of ODSH, as shown in Figure 3A–D (wet/dry lung ratio). The concurrent reduction in BALF IL-6 and TNF-α levels highlights ODSH’s ability to limit the severity of the acute cytokine response, which is central to the pathogenesis of LPS-induced lung injury [53,54]. 

Beyond these physiological outcomes, a central observation of this study is the broad suppression of pro-inflammatory gene expression by ODSH. LPS exposure markedly increased transcripts associated with cytokine production, MAPK activation, nitric oxide synthesis, and leukocyte adhesion [55,56,57]. ODSH consistently attenuated these elevations across HLMVECs and mouse lungs, indicating that its anti-inflammatory actions extend beyond individual mediators to encompass multiple nodes of the inflammatory cascade. The reduction in IL-1β, TNF-α, IL-6, NOS2, MAPK14, MAPK1, ICAM1, and CCL2 suggests that ODSH modulates pathways involved in endothelial activation and leukocyte recruitment.

In parallel, ODSH strengthened anti-inflammatory signaling, as reflected by enhanced IL-10 expression. IL-10 functions as a key counter-regulatory mediator that restrains excessive inflammation and promotes resolution [58,59,60]. Its modulation by ODSH indicates that the compound may enhance endogenous counter-regulatory mechanisms that contribute to immune homeostasis. This dual regulatory action likely contributes to the overall reduction in lung injury observed in vivo.

**Figure 11:**
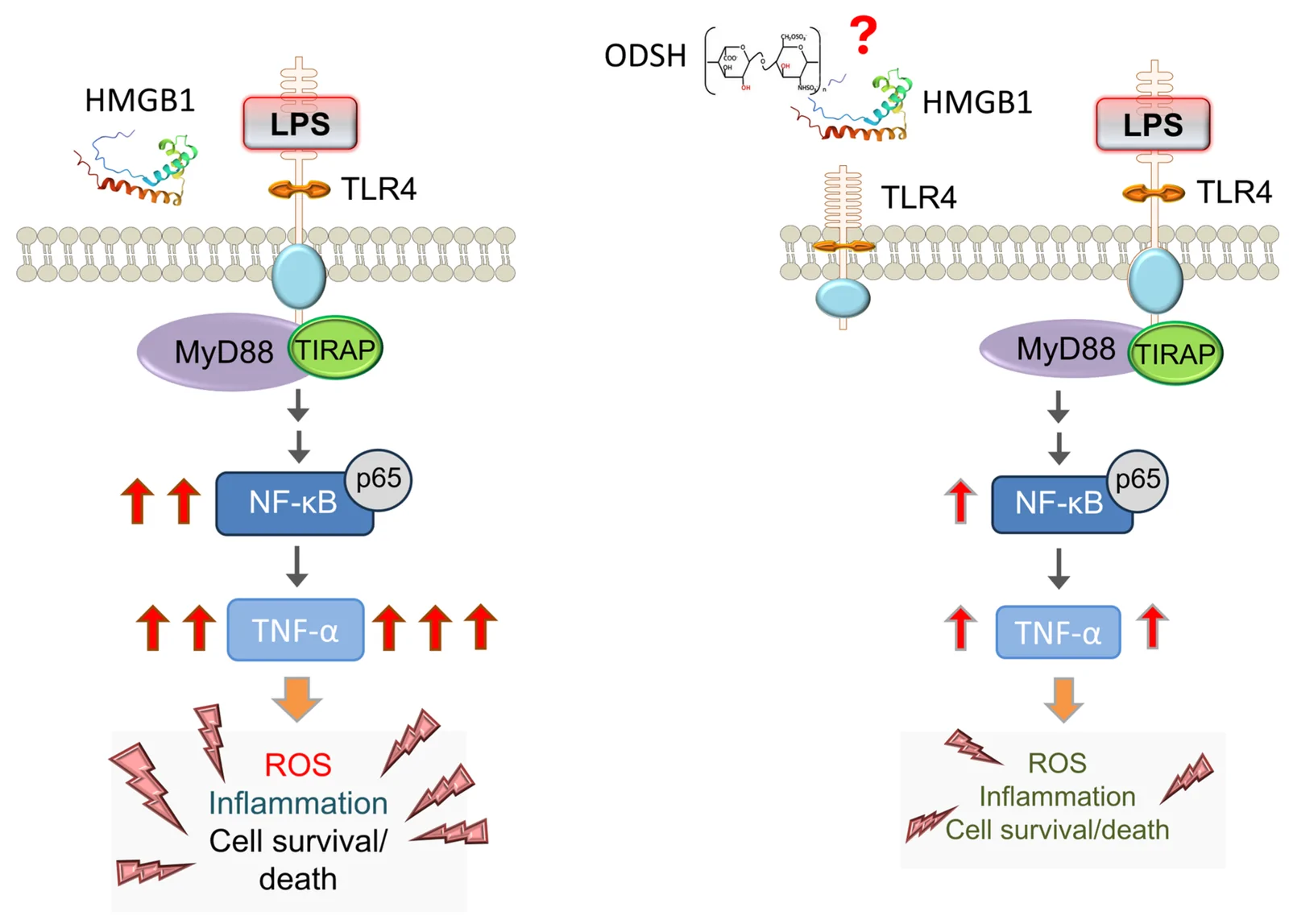
Proposed mechanism of ODSH-mediated endothelial protection in LPS-induced acute lung injury. Under inflammatory stimulation, LPS and HMGB1 engage TLR4, recruiting MyD88 and TIRAP, which activate NF-κB (p65) and upregulate TNF-α, leading to increased reactive oxygen species (ROS) generation, inflammation, and endothelial injury. Prior studies have reported interactions between ODSH and HMGB1, which may contribute to the modulation of inflammatory signaling. These mechanisms were not directly investigated in the present study and are included as literature-supported hypotheses.

The integrity of the endothelial barrier is a critical determinant of ALI severity [43], and our data show that ODSH preserves this barrier at the transcriptional level. An LPS-induced downregulation of CDH5, OCLN, CLDN5, and TJP1 [61], key components of adherens and tight junctions, was reversed by ODSH. The restoration of these junctional genes aligns with our earlier findings of reduced EBDA leakage and improved wet-to-dry ratios, supporting the concept that ODSH supports the preservation of endothelial barrier-associated transcriptional programs during inflammatory stress. Importantly, ODSH also modulated components of innate immune signaling, including the TLR4–MyD88–NF-κB axis. An LPS-induced upregulation of TLR4, MYD88, and RELA [62] was significantly attenuated by ODSH, indicating interference with the early recognition and transduction of LPS signals. These transcriptional findings were validated at the protein level in both HLMVECs and mouse lung lysates, where ODSH reduced the abundance of NF-κB, MyD88, and TNF-α. Together, these findings suggest that ODSH modulates inflammatory responses at multiple transcriptional and protein-expression levels (a possible mechanism is outlined in Figure 11). Our observations align with established evidence that cytokines such as TNF-α and IL-6 amplify endothelial activation by engaging NF-κB-driven inflammatory pathways, which is a process broadly implicated in vascular dysfunction and inflammatory disease [63,64].

The combined effects of ODSH on endothelial barrier genes, inflammatory mediators, and innate immune signaling provide a mechanistic framework that explains its protective actions in LPS-induced ALI. By simultaneously suppressing pro-inflammatory pathways and enhancing anti-inflammatory responses, ODSH shifts the balance toward resolution rather than the propagation of injury. These findings complement our previously published work [36] showing reduced vascular permeability, neutrophil infiltration, and pro-inflammatory signaling in vivo, and they further support the therapeutic potential of ODSH as a modulator of endothelial and immune responses in ALI. Below, we provide a schematic representation summarizing the experimentally observed findings together with potential mechanisms proposed in the literature [65,66,67,68,69]. 

It is worth noting that in addition to our observed effects of ODSH on TLR4 expression, ODSH may affect LPS-induced pro-inflammatory signaling through binding with RAGE (Receptor for Advanced Glycation Endproducts). LPS activates both TLR4 and RAGE, and both RAGE and TLR4 are HMGB1 receptors [70,71]. The anti-inflammatory effects of ODSH may also involve the disruption of HMGB1–RAGE signaling, as ODSH has been reported to bind HMGB1 and inhibit RAGE–ligand interactions [26]. The HMGB1–RAGE axis is involved in apoptotic injury during ARDS [72]. Further, newly published data demonstrated that RAGE, but not TLR4, mediates LPS-induced skin inflammatory pain in humans [73]. However, recent findings also demonstrated a crosstalk between RAGE and TLR4 signaling in LPS/high glucose-induced MyD88/NF-κB pathway activation and the release of pro-inflammatory cytokines in macrophages [70]. The role of ODSH in suppressing RAGE-mediated mechanisms of LPS-induced ALI requires further investigation. Recent studies have further highlighted the importance of HMGB1-, MD2-, TLR4-, and RAGE-dependent signaling pathways in the pathogenesis of acute inflammatory injury. The modulation of HMGB1/NF-κB [65,66], HMGB1/TLR4/NF-κB [67,68], HMGB1/MyD88/NF-κB [69], HMGB1/RAGE/NF-κB [74], HMGB1/MD2/TLR4 [75], and MD2/TLR4 signaling pathways [76,77] has been shown to attenuate inflammatory responses and tissue injury in multiple experimental models. These observations support the possibility that ODSH-mediated protection may involve the modulation of HMGB1-associated signaling networks; however, the direct investigation of these mechanisms was beyond the scope of the present study. 

Collectively, this study reinforces the concept that ODSH exerts multifaceted protective effects in LPS-induced lung injury. Its ability to regulate inflammatory signaling, preserve endothelial junctional integrity, and attenuate innate immune activation supports the continued mechanistic and preclinical evaluation of ODSH in the treatment of ALI/ARDS and related inflammatory lung disorders.

### Limitations and Future Directions

Although the present study provides substantial mechanistic insight into the protective actions of ODSH, we would like to acknowledge several limitations that warrant consideration. Our current work primarily focused on downstream inflammatory and barrier-regulatory pathways, and the precise upstream molecular interactions through which ODSH exerts its effects remain to be fully defined. In particular, the potential involvement of HMGB1-, MD2-, and RAGE-dependent signaling was not directly examined despite their established roles in amplifying LPS-mediated inflammation and acute lung injury [78,79]. Considering these limitations, future studies can be undertaken to investigate whether ODSH interferes with these ligand–receptor interactions or modulates their downstream adaptor complexes. Parallelly, junctional proteins including VE-cadherin, occludin, claudin-5, and ZO-1 were evaluated only at the transcriptional level in this study. Future studies should assess protein abundance and localization to further define the mechanisms underlying ODSH-mediated barrier protection. We acknowledge the relatively small sample size used in the present proof-of-concept study. Future investigations employing larger cohorts are warranted to further validate these findings. Additionally, while the current findings demonstrate robust protection in vitro and in a murine ALI model, further validation in additional preclinical models (clinically relevant injury models as well) is needed to assess the translational potential of ODSH. Future studies should also explore optimized delivery approaches and lung-targeted therapeutic strategies to maximize efficacy while minimizing systemic effects [80]. Importantly, comprehensive pharmacokinetic, dosing, and safety studies will also be essential to determine its suitability for therapeutic development. The present study complements that work by providing direct experimental evidence that ODSH preserves endothelial barrier function through modulation of TLR4–MyD88–NF-κB signaling in LPS-induced acute lung injury.

## 5. Conclusions

As evidenced, ODSH preserves endothelial barrier function in LPS-challenged HLMVECs, as shown by sustained TER measurements. ODSH also reduced BALF protein levels, inflammatory cell counts, EBDA leakage, and the wet/dry ratio in mouse lung tissue. Further, ODSH downregulates pro-inflammatory gene expression and upregulates barrier-stabilizing and anti-inflammatory markers both in HLMVECs and mouse lung tissue. Our findings demonstrate that ODSH preserves endothelial barrier function and modulates inflammatory responses associated with TLR4–MyD88–NF-κB signaling in experimental ALI, providing additional mechanistic insight into ODSH-mediated protection. By modulating the inflammatory responses associated with these pathways, ODSH exhibits potential therapeutic value by enhancing barrier integrity and attenuating inflammatory signaling, supporting its relevance for further mechanistic and preclinical investigation in ALI and ARDS.

## Data Availability

The original contributions presented in the study are included in the article; further inquiries can be directed at the corresponding author.

## Supplementary Materials

The following supporting information can be downloaded at https://www.mdpi.com/article/10.3390/biom16101516/s1, Table S1: Primer sequences used for RT-qPCR analysis in the human cell (HLMVECs) study; Table S2: Primer sequences used for RT-qPCR analysis in mice (CD-1) lungs study; Figure S1: Western blot images show the expression levels of (A) NF-κB and MyD88, (B) NF-κB, MyD88, and TNF-α in HLMVECs; Figure S2: Protein expression of NF-κB, MyD88, and TNF-α was examined by Western blotting using mouse lung lysates.

## Abbreviations

The following abbreviations are used in this manuscript:

ACK	Ammonium–chloride–potassium
ALI	Acute lung injury
AML	Acute myeloid leukemia
ARDS	Acute respiratory distress syndrome
BALF	Bronchoalveolar lavage fluid
BCA	Bicinchoninic acid
BSA	Bovine serum albumin
CCL2	C-C motif chemokine ligand 2
CDH5	Cadherin-5 (VE-cadherin)
CLDN5	Claudin-5
DMSO	Dimethyl sulfoxide
EBDA	Evans blue dye albumin
ECIS	Electric cell-substrate impedance sensing
ECL	Enhanced chemiluminescence
EDTA	Ethylenediaminetetraacetic acid
ELISA	Enzyme-linked immunosorbent assay
EU	Endotoxin unit
HBSS	Hanks’ balanced salt solution
HLMVECs	Human lung microvascular endothelial cells
HMGB1	High mobility group box 1
HPRT1	Hypoxanthine phosphoribosyltransferase 1
HRP-conjugated	Horseradish peroxidase–conjugated
ICAM1	Intercellular adhesion molecule 1
IJV	Internal jugular vein
IL-1B	Interleukin-1 beta
IL-6	Interleukin-6
IL-10	Interleukin-10
IT	Intratracheal
LPS	Lipopolysaccharide
MAPK1	Mitogen-activated protein kinase 1
MAPK14	Mitogen-activated protein kinase 14 (p38α)
MTT	3-(4,5-Dimethylthiazol-2-yl)-2,5-diphenyltetrazolium bromide
MyD88	Myeloid differentiation primary response 88
NF-κB	Nuclear factor kappa-light-chain-enhancer of activated B cells
NOS2	Nitric oxide synthase 2
OCLN	Occludin
ODSH	2-O, 3-O desulfated heparin
PF4	Platelet factor 4
PBS	Phosphate-buffered saline
PVDF	Polyvinylidene difluoride membranes
RAGE	Receptor for advanced glycation end-products
RELA	NF-κB p65 subunit
RT-qPCR	Reverse transcription quantitative polymerase chain reaction
SDS–PAGE	Sodium dodecyl sulfate–polyacrylamide gel electrophoresis
SEM	Standard error of the mean
SD	Standard deviation
TER	Transendothelial electrical resistance
TJP1	Tight junction protein 1
TLR4	Toll-like receptor 4
TNF-α	Tumor necrosis factor alpha
W/D	Wet-to-dry lung weight ratio
ZO-1	Zonula occludens-1
