# Supplementary material for: 2-O, 3-O Desulfated Heparin (ODSH) Preserves Endothelial Barrier Integrity in Lipopolysaccharide-Induced Acute Lung Injury by Modulating TLR4–MyD88–NF-κB Signaling
Source: Biomolecules. 2026 Oct 18;16(10):1516. doi: 10.3390/biom16101516 (PMC13702097; doi:10.3390/biom16101516)
Supplement: Supplementary file 1 [file biomolecules-16-01516-s001.zip › biomolecules-4607153-origin WB.pdf]

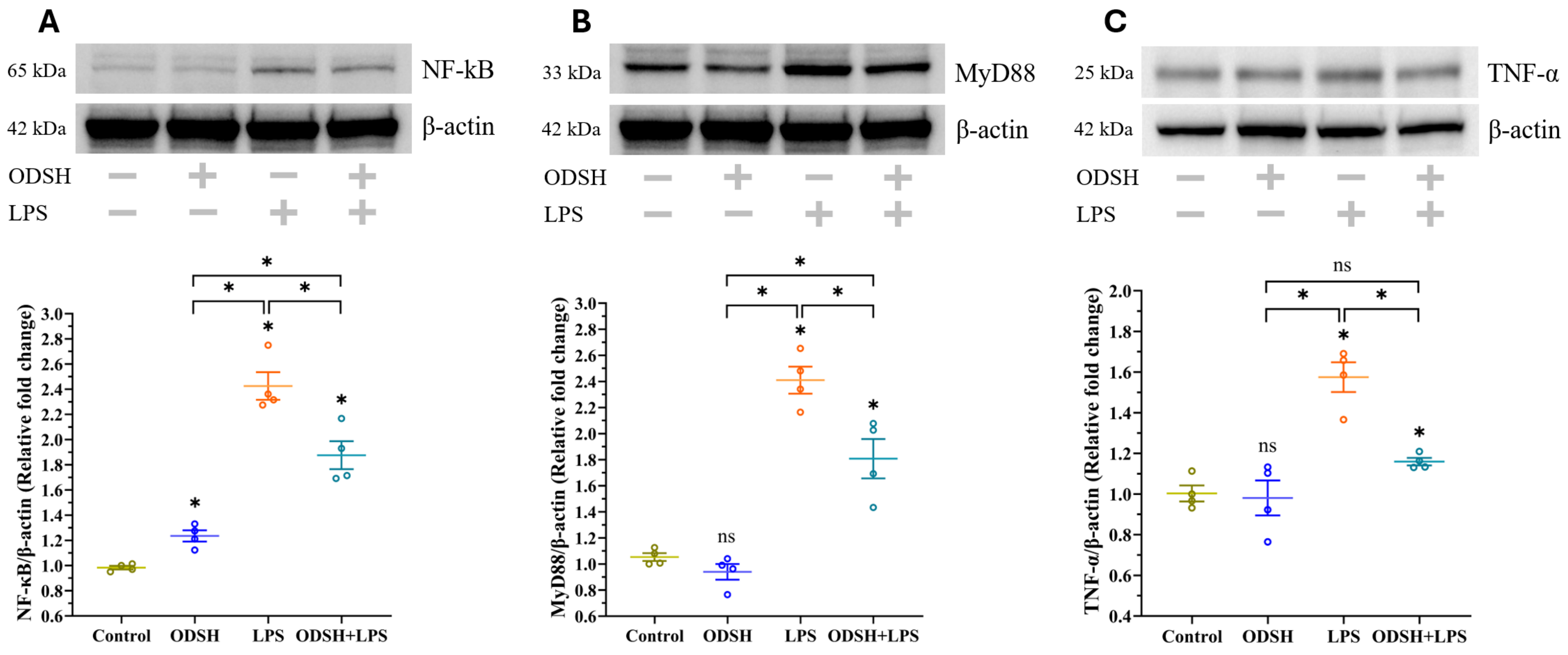
A
65 kDa
NF-kB
42 kDa
β-actin
ODSH
LPS
NF-κB/β-actin (Relative fold change)
Control
ODSH
LPS
ODSH+LPS
B
33 kDa
MyD88
42 kDa
β-actin
ODSH
LPS
MyD88/β-actin (Relative fold change)
ns
Control
ODSH
LPS
ODSH+LPS
C
25 kDa
TNF-α
42 kDa
β-actin
ODSH
LPS
TNF-α/β-actin (Relative fold change)
ns
Control
ODSH
LPS
ODSH+LPS

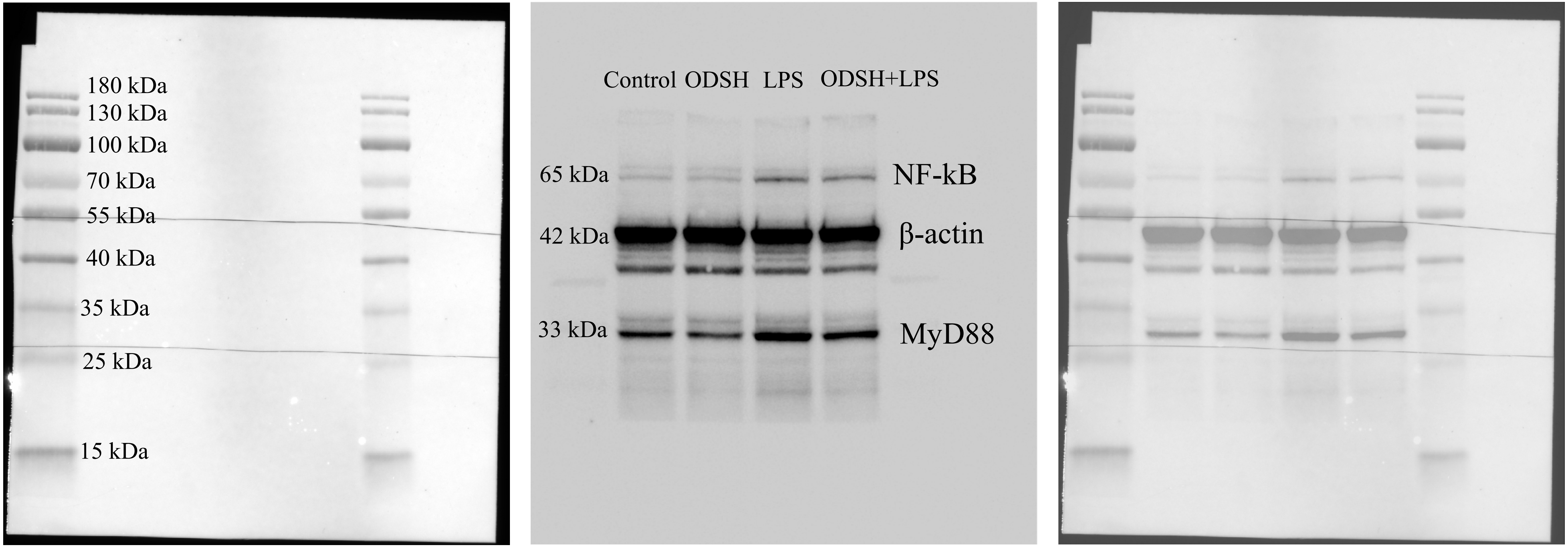
180 kDa
130 kDa
100 kDa
70 kDa
55 kDa
40 kDa
35 kDa
25 kDa
15 kDa
Control
ODSH
LPS
ODSH+LPS
65 kDa
42 kDa
33 kDa
NF-kB
β-actin
MyD88

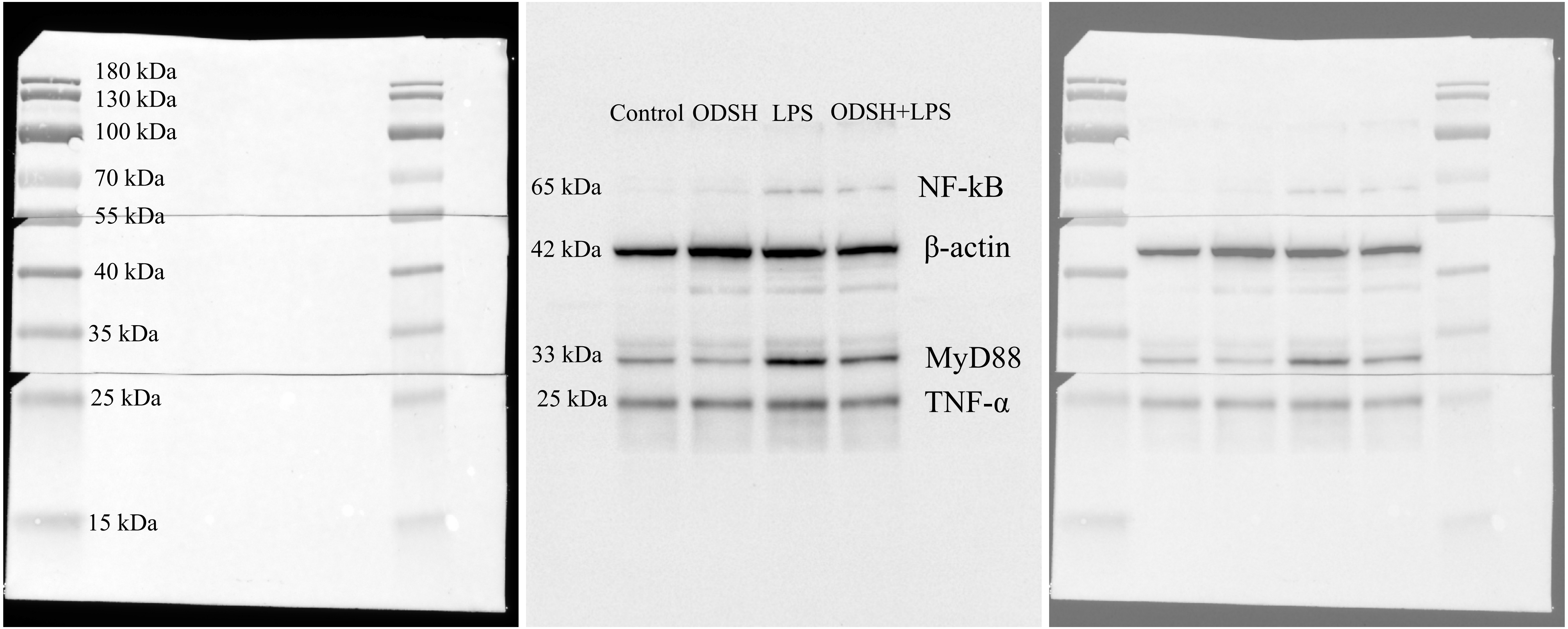
180 kDa
130 kDa
100 kDa
70 kDa
55 kDa
40 kDa
35 kDa
25 kDa
15 kDa
Control
ODSH
LPS
ODSH+LPS
65 kDa
42 kDa
33 kDa
25 kDa
NF-kB
β-actin
MyD88
TNF-α

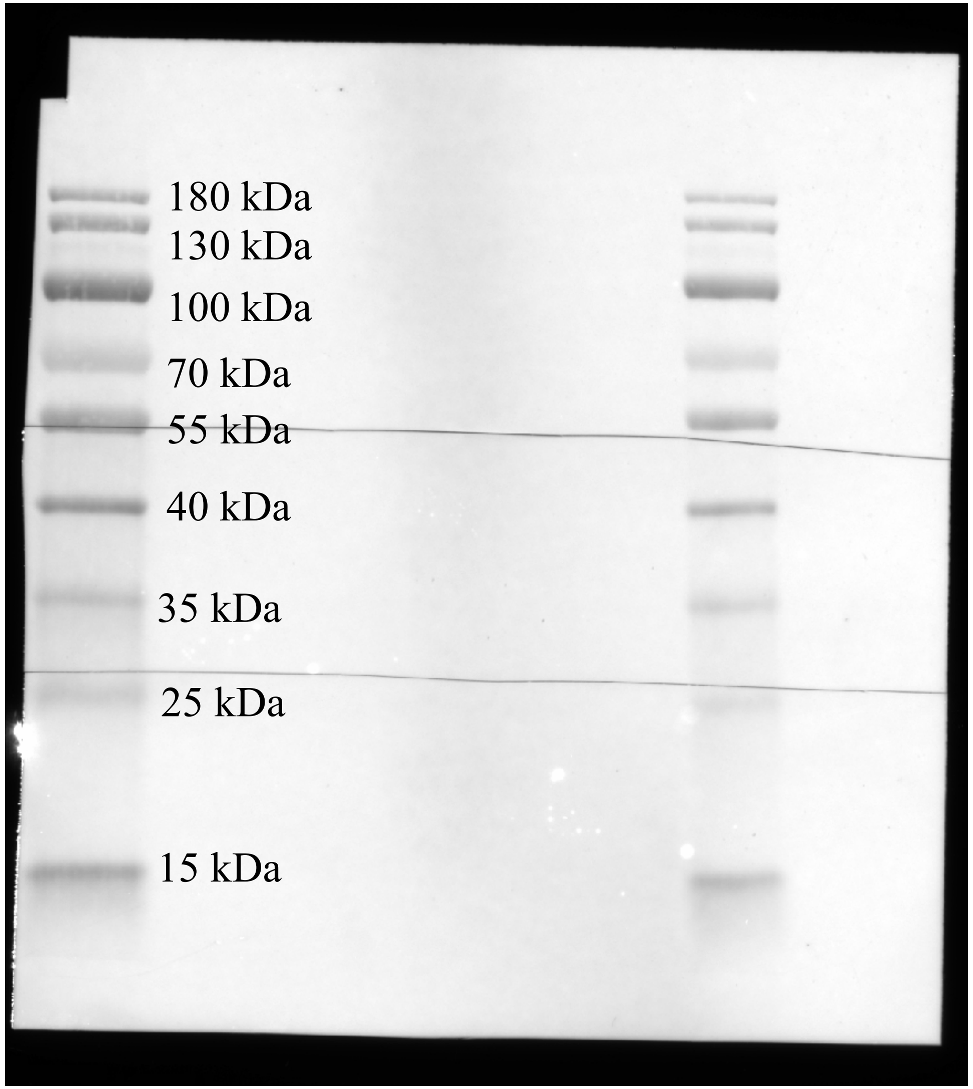
180 kDa
130 kDa
100 kDa
70 kDa
55 kDa
40 kDa
35 kDa
25 kDa
15 kDa

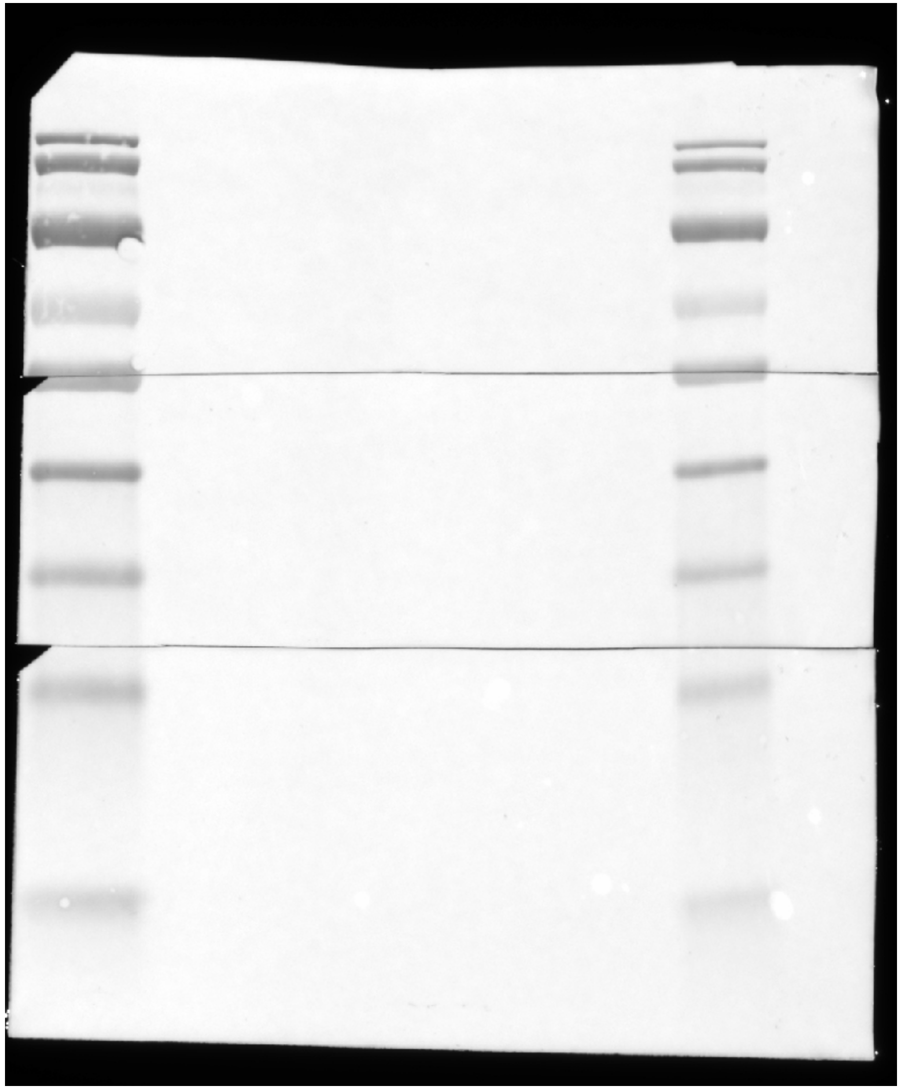

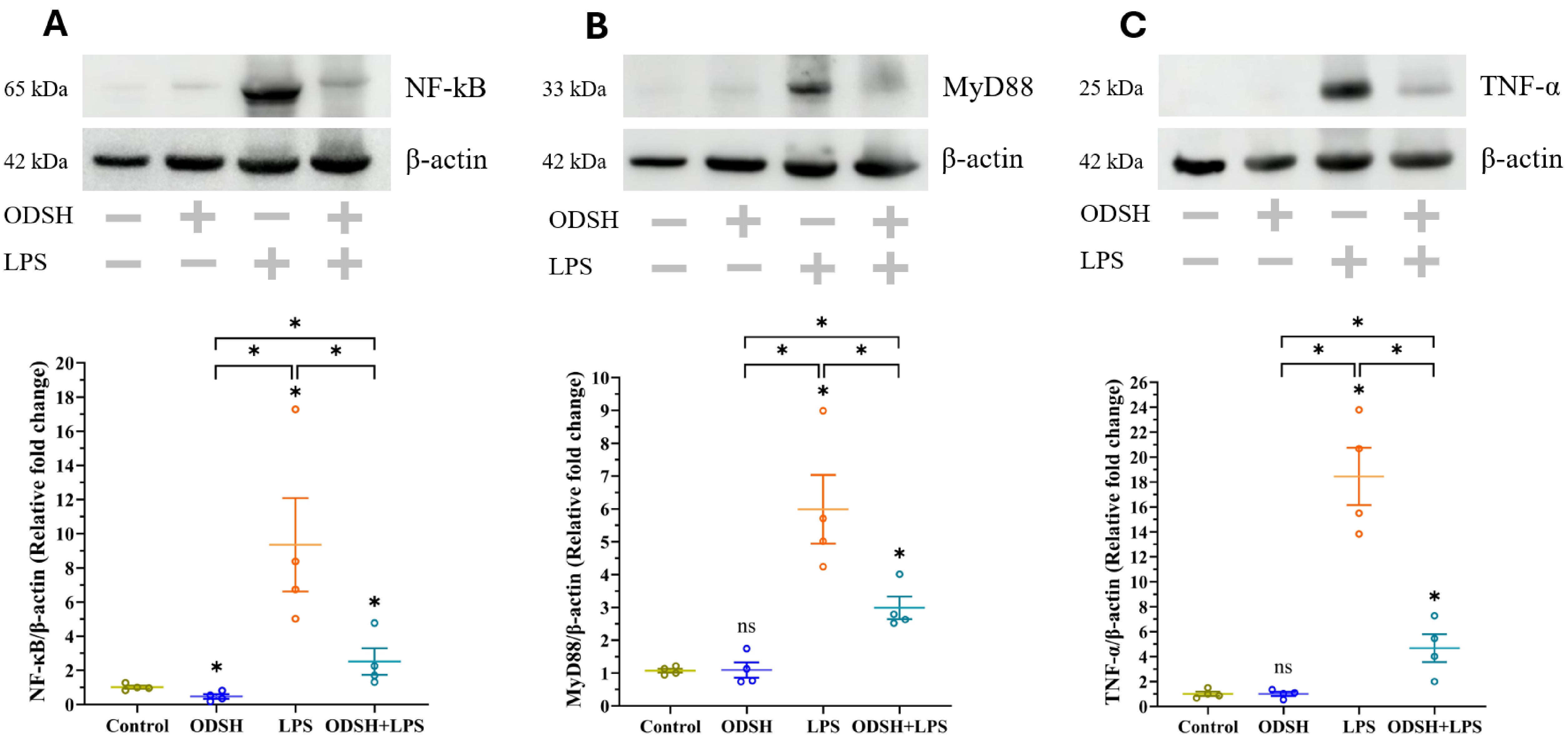
A
65 kDa
NF-kB
42 kDa
β-actin
ODSH
LPS
NF-κB/β-actin (Relative fold change)
Control
ODSH
LPS
ODSH+LPS
B
33 kDa
MyD88
42 kDa
β-actin
ODSH
LPS
MyD88/β-actin (Relative fold change)
ns
Control
ODSH
LPS
ODSH+LPS
C
25 kDa
TNF-α
42 kDa
β-actin
ODSH
LPS
TNF-α/β-actin (Relative fold change)
ns
Control
ODSH
LPS
ODSH+LPS

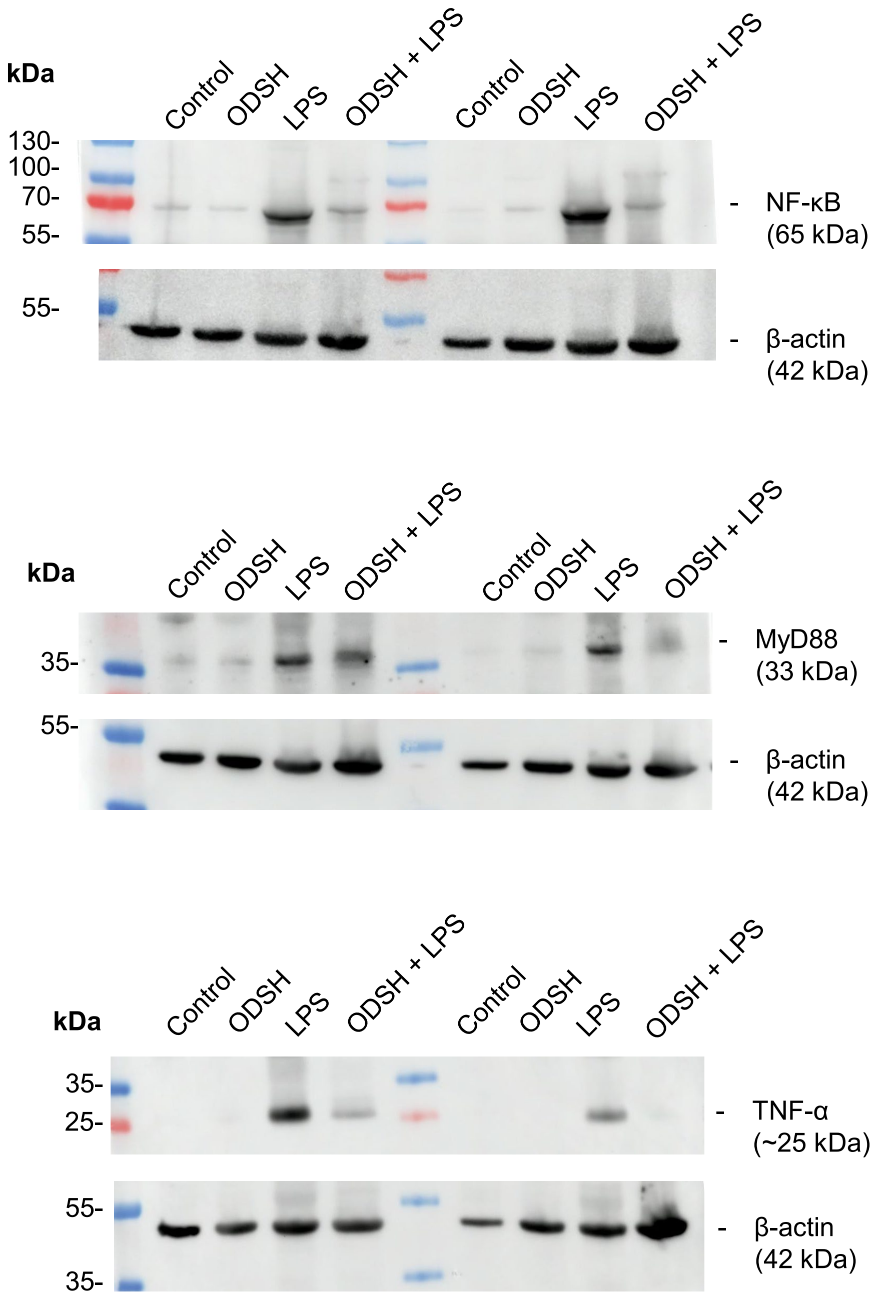

kDa
Control
ODSH
LPS
ODSH + LPS
Control
ODSH
LPS
ODSH + LPS
130-
100-
70-
55-
NF-κB
(65 kDa)
55-
β-actin
(42 kDa)
kDa
Control
ODSH
LPS
ODSH + LPS
Control
ODSH
LPS
ODSH + LPS
35-
MyD88
(33 kDa)
55-
β-actin
(42 kDa)
kDa
Control
ODSH
LPS
ODSH + LPS
Control
ODSH
LPS
ODSH + LPS
35-
25-
TNF-α
(~25 kDa)
55-
35-
β-actin
(42 kDa)

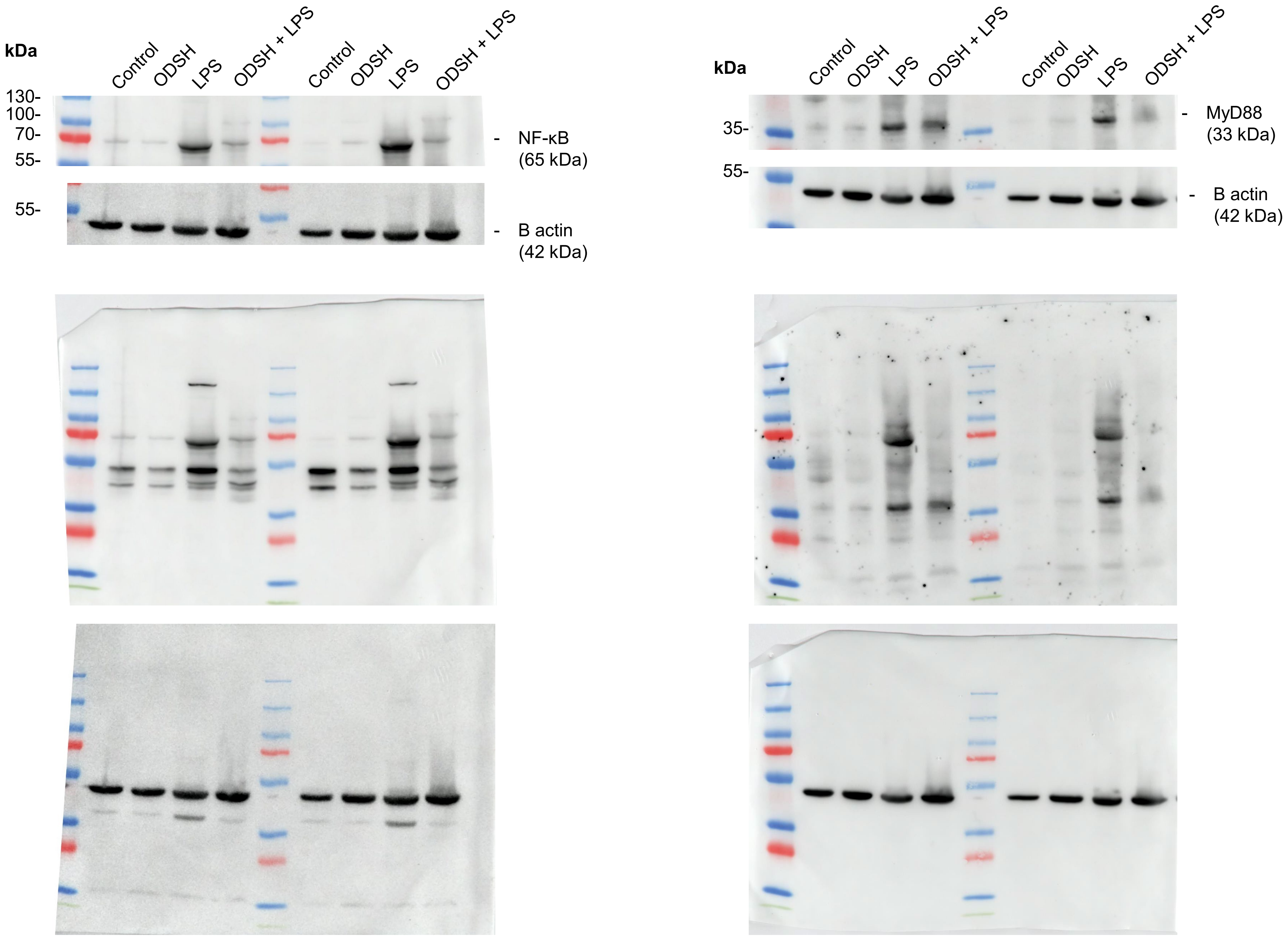

kDa
Control
ODSH
LPS
ODSH + LPS
Control
ODSH
LPS
ODSH + LPS
130-
100-
70-
55-
NF-κB
(65 kDa)
55-
B actin
(42 kDa)
kDa
Control
ODSH
LPS
ODSH + LPS
Control
ODSH
LPS
ODSH + LPS
35-
MyD88
(33 kDa)
55-
B actin
(42 kDa)

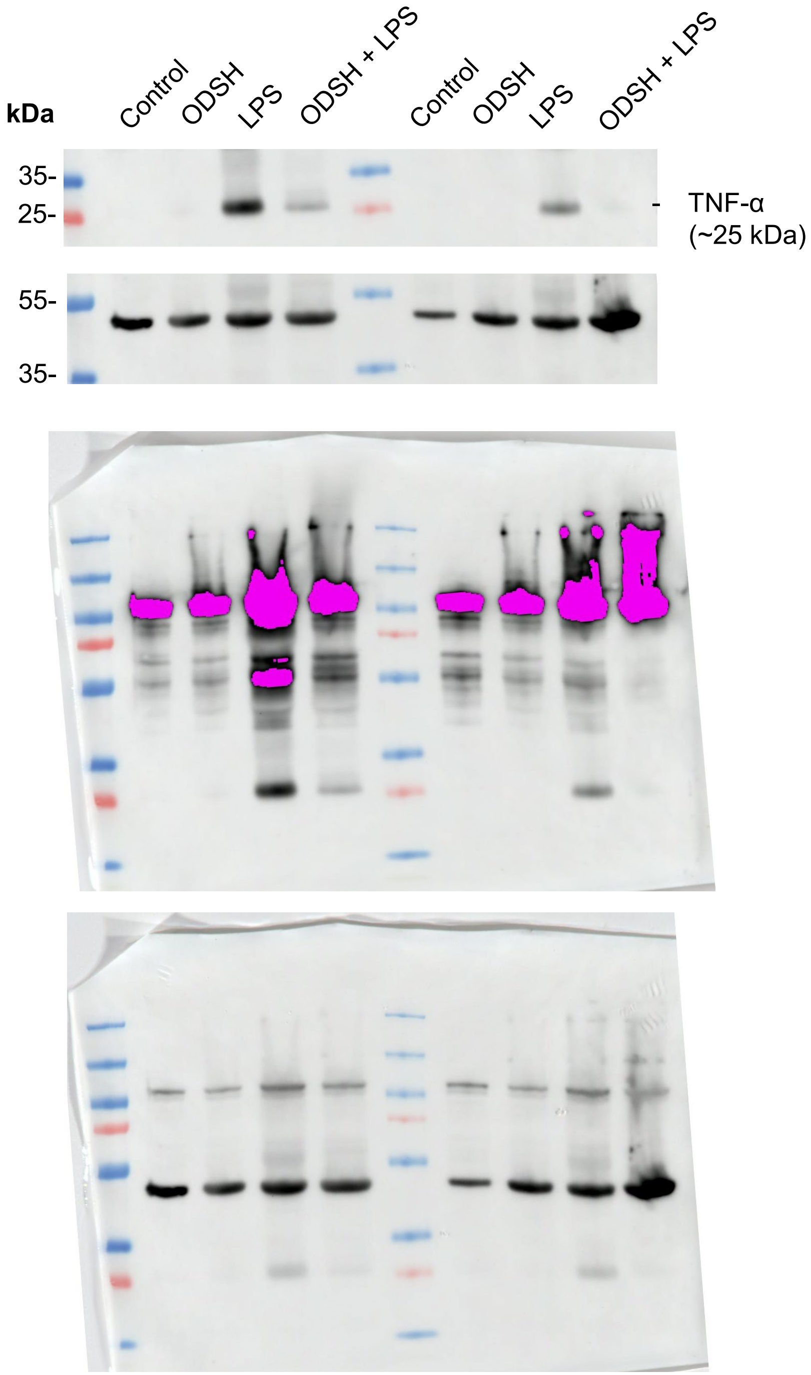
kDa
Control
ODSH
LPS
ODSH + LPS
Control
ODSH
LPS
ODSH + LPS
35-
25-
TNF-α
(~25 kDa)
55-
35-
