# Supplementary material for: 2-O, 3-O Desulfated Heparin (ODSH) Preserves Endothelial Barrier Integrity in Lipopolysaccharide-Induced Acute Lung Injury by Modulating TLR4–MyD88–NF-κB Signaling
Source: Biomolecules. 2026 Oct 18;16(10):1516. doi: 10.3390/biom16101516 (PMC13702097; doi:10.3390/biom16101516)
Supplement: Supplementary file 1 [file biomolecules-16-01516-s001.zip › biomolecules-4607153-supplementary.pdf]

Table S1: Primer sequences used for RT-qPCR analysis in the human cell (HLMVECs) study

**Gene name**	**5′→3′ Forward primer sequence**	**5′→3′ Reverse primer sequence**	**OriGene ID**
HPRT1	CATTATGCTGAGGATTTGGAAAGG	CTTGAGCACACAGAGGGCTACA	NM_000194
IL1β	CCACAGACCTTCCAGGAGAATG	GTGCAGTTCAGTGATCGTACAGG	NM_000576
TNFA	CTCTTCTGCCTGCTGCACTTTG	ATGGGCTACAGGCTTGTCACTC	NM_000594
IL6	AGACAGCCACTCACCTCTTCAG	TTCTGCCAGTGCCTCTTTGCTG	NM_000600
RELA	TGAACCGAAACTCTGGCAGCTG	CATCAGCTTGCGAAAAGGAGCC	NM_021975
NOS2	GCTCTACACCTCCAATGTGACC	CTGCCGAGATTTGAGCCTCATG	NM_000625
MAPK14	GAGCGTTACCAGAACCTGTCTC	AGTAACCGCAGTTCTCTGTAGGT	NM_001315
MAPK1	ACACCAACCTCTCGTACATCGG	TGGCAGTAGGTCTGGTGCTCAA	NM_002745
ICAM1	AGCGGCTGACGTGTGCAGTAAT	TCTGAGACCTCTGGCTTCGTCA	NM_000201
CCL2	AGAATCACCAGCAGCAAGTGTCC	TCCTGAACCCACTTCTGCTTGG	NM_002982
IL10	TCTCCGAGATGCCTTCAGCAGA	TCAGACAAGGCTTGGCAACCCA	NM_000572
CDH5	GAAGCCTCTGATTGGCACAGTG	TTTTGTGACTCGGAAGAACTGGC	NM_001795
OCLN	ATGGCAAAGTGAATGACAAGCGG	CTGTAACGAGGCTGCCTGAAGT	NM_002538
CLDN5	ATGTGGCAGGTGACCGCCTTC	CGAGTCGTACACTTTGCACTGC	NM_003277
TJP1	GTCCAGAATCTCGGAAAAGTGCC	CTTTCAGCGCACCATACCAACC	NM_003257
TLR4	CCCTGAGGCATTTAGGCAGCTA	AGGTAGAGAGGTGGCTTAGGCT	NM_138554
MYD88	GAGGCTGAGAAGCCTTTACAGG	GCAGATGAAGGCATCGAAACGC	NM_002468

Table S2: Primer sequences used for RT-qPCR analysis in mice (CD-1) lungs study

**Gene name**	**5′→3′ Forward primer sequence**	**5′→3′ Reverse primer sequence**	**OriGene ID**
Hprt1	CTGGTGAAAAGGACCTCTCGAAG	CCAGTTTCACTAATGACACAAACG	NM_013556
Il1b	TGGACCTTCCAGGATGAGGACA	GTTCATCTCGGAGCCTGTAGTG	NM_008361
Tnf	GGTGCCTATGTCTCAGCCTCTT	GCCATAGAACTGATGAGAGGGAG	NM_013693
Il6	TACCACTTCACAAGTCGGAGGC	CTGCAAGTGCATCATCGTTGTTC	NM_031168
Rela	TCCTGTTCGAGTCTCCATGCAG	GGTCTCATAGGTCCTTTTGCGC	NM_009045
Nos2	GAGACAGGGAAGTCTGAAGCAC	CCAGCAGTAGTTGCTCCTCTTC	NM_010927
Mapk14	CCGAACGATACCAGAACCTGTC	ACGCAACTCTCGGTAGGTCCTT	NM_011951
Mapk1	TCAAGCCTTCCAACCTCCTGCT	AGCTCTGTACCAACGTGTGGCT	NM_001038663
Icam1	AAACCAGACCCTGGAACTGCAC	GCCTGGCATTTCAGAGTCTGCT	NM_010493
Ccl2	GCTACAAGAGGATCACCAGCAG	GTCTGGACCCATTCCTTCTTGG	NM_011333
Il10	CGGGAAGACAATAACTGCACCC	CGGTTAGCAGTATGTTGTCCAGC	NM_010548
Cdh5	GAACGAGGACAGCAACTTCACC	GTTAGCGTGCTGGTTCCAGTCA	NM_009868
Ocln	TGGCAAGCGATCATACCCAGAG	CTGCCTGAAGTCATCCACACTC	NM_008756
Cldn5	TGACTGCCTTCCTGGACCACAA	CATACACCTTGCACTGCATGTGC	NM_013805
Tjp1	GTTGGTACGGTGCCCTGAAAGA	GCTGACAGGTAGGACAGACGAT	NM_009386
Tlr4	AGCTTCTCCAATTTTTCAGAACTTC	TGAGAGGTGGTGTAAGCCATGC	NM_021297
Myd88	ACCTGTGTCTGGTCCATTGCCA	GCTGAGTGCAAACTTGGTCTGG	NM_010851

Primers used for human and mouse RT-qPCR assays were referenced from OriGene Technologies, Inc. (USA) and synthesized by Integrated DNA Technologies (IDT, Coralville, IA, USA).

PCR amplification consisted of an initial incubation at 50 °C for 2 min followed by 95 °C for 2 min, then 40 cycles of denaturation at 95 °C for 15 s, annealing at 60 °C for 15 s, and extension at 72 °C for 1 min.

Dissociation curve analysis was performed with sequential steps at 95 °C for 15 s, 60 °C for 1 min, and 95 °C for 15 s.

[A]

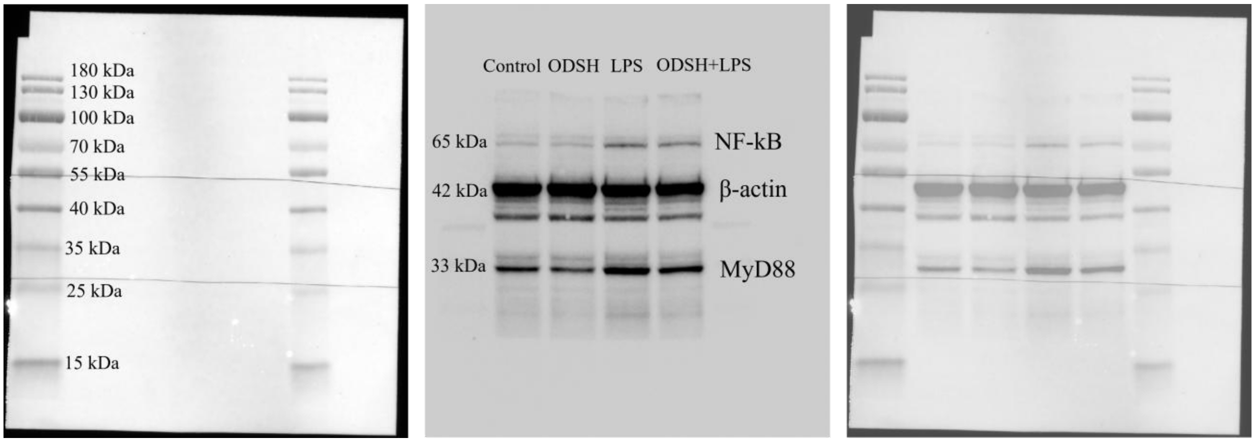

[B]

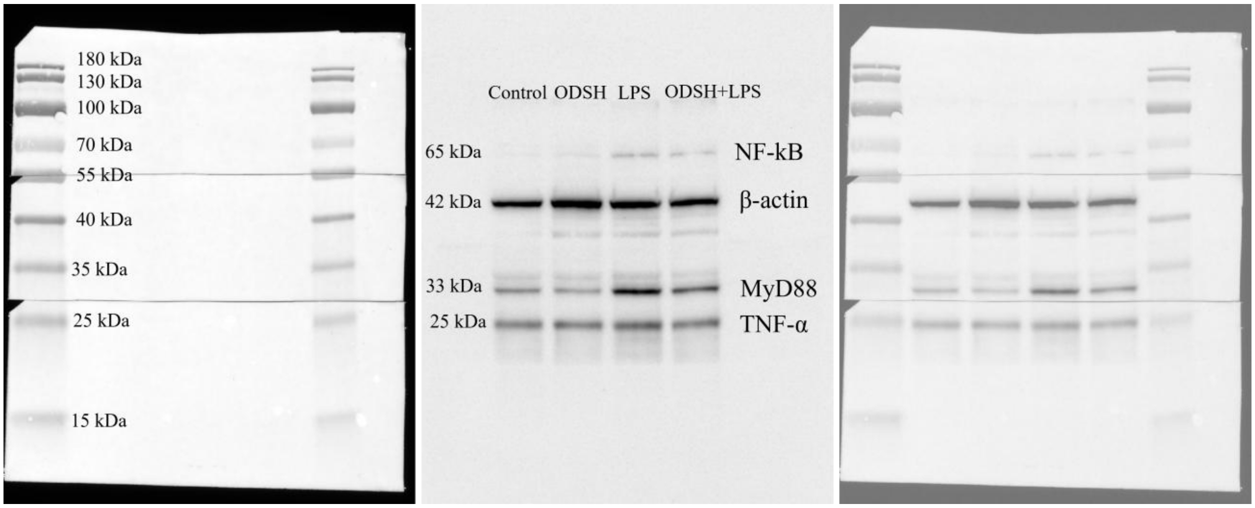

Figure S1: Western blot images show the expression levels of (**A**) NF-κB, MyD88, (**B**) NF-κB, MyD88, and TNF-α, with β-actin used as the loading control to confirm equal protein input across the samples extracted from HLMVECs using lysis buffer (2X Laemmli buffer + protease inhibitor + phosphatase inhibitor + β-Mercaptoethanol). For each group, 15 µg of protein was loaded, all derived from the same aliquot to maintain consistency between control and experimental samples.

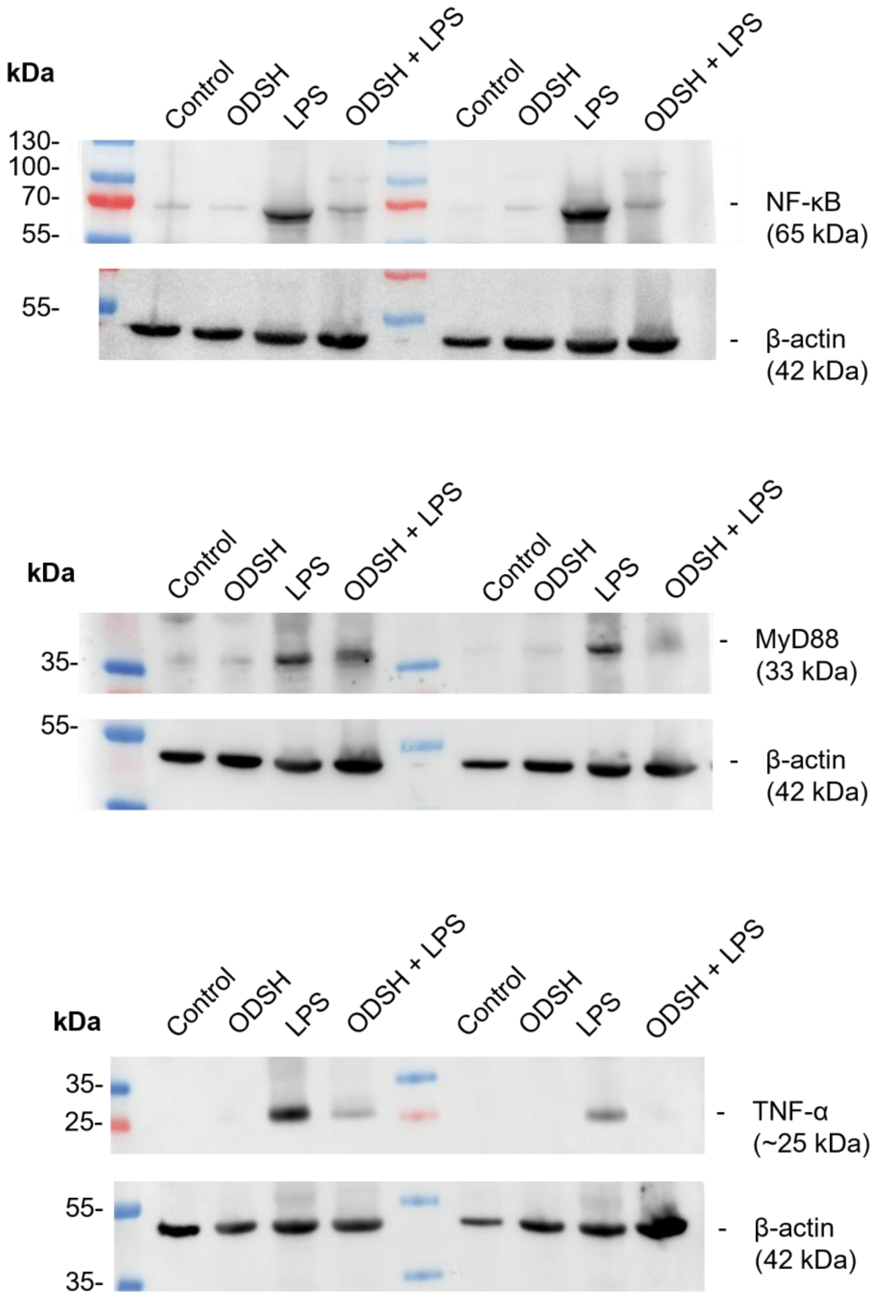

Figure S2: Protein expression of NF-κB, MyD88, and TNF-α was examined by Western blotting using mouse lung lysates from the experimental groups. β-actin was used as the loading control to confirm uniform protein loading. A total of 10 µg of protein per lane was applied, with all samples drawn from a same aliquot to maintain comparability across experimental groups.
